# High frequency of +1 programmed ribosomal frameshifting in *Euplotes octocarinatus*

**DOI:** 10.1038/srep21139

**Published:** 2016-02-19

**Authors:** Ruanlin Wang, Jie Xiong, Wei Wang, Wei Miao, Aihua Liang

**Affiliations:** 1 Key Laboratory of Chemical Biology and Molecular Engineering of Ministry of Education, Institute of Biotechnology, Shanxi University, Taiyuan 030006, China; 2 Key Laboratory of Aquatic Biodiversity and Conservation, Institute of Hydrobiology, Chinese Academy of Sciences, Wuhan 430072, China

## Abstract

Programmed −1 ribosomal frameshifting (−1 PRF) has been identified as a mechanism to regulate the expression of many viral genes and some cellular genes. The slippery site of −1 PRF has been well characterized, whereas the +1 PRF signal and the mechanism involved in +1 PRF remain poorly understood. Previous study confirmed that +1 PRF is required for the synthesis of protein products in several genes of ciliates from the genus *Euplotes*. To accurately assess the frequency of genes requiring frameshift in *Euplotes*, the macronuclear genome and transcriptome of *Euplotes octocarinatus* were analyzed in this study. A total of 3,700 +1 PRF candidate genes were identified from 32,353 transcripts, and the gene products of these putative +1 PRFs were mainly identified as protein kinases. Furthermore, we reported a putative suppressor tRNA of UAA which may provide new insights into the mechanism of +1 PRF in euplotids. For the first time, our transcriptome-wide survey of +1 PRF in *E. octocarinatus* provided a dataset which serves as a valuable resource for the future understanding of the mechanism underlying +1 PRF.

As a typical ciliate, *Euplotes octocarinatus* exhibits nuclear dimorphism [micronucleus (MIC) and macronucleus (MAC)]. The MIC is diploid and transcriptionally inert during most of its life cycle, enabling the transmission of genetic information between generations by sexual reproduction. The MAC is considered the somatic nucleus, which is transcriptionally active during the vegetative growth^1^. During conjugation, the MAC is degraded and a new MAC is developed from the zygote nucleus accompanied by DNA rearrangements. Similar to that of other hypotrichous ciliates, the MAC of *E. octocarinatus* contains abundant gene-sized DNA molecules (‘nanochromosome’, with mean length ~2 kb), each of which is differentially amplified^2^. All nanochromosomes have telomeric repeats 5′-(C_4_A_4_)_n_-3′ at their ends^3^.

The following unique features distinguish *Euplotes* from other ciliates: 1) the conventional stop codon UGA is reassigned as cysteine^4^ or selenocysteine^5^, which means that only the UAA and UAG are used as stop codons in *Euplotes*; and 2) the high frequency of +1 programmed ribosomal frameshifting (PRF) in *Euplotes*^6^. PRF is a recoding event by which the translating ribosome switches from the initial (0) reading frame to the −1 or +1 reading frame at a specific position, and then continues its translation^7^. Although the first reported frameshifting sequence has been found in viruses, it is becoming increasingly apparent that PRF is also widespread and likely exists in all branches of life from bacteria to higher eukaryotes^8^,^9^,^10^.

On the basis of the reading frame shift, two main PRFs (−1 and +1) were reported in viruses and other cellular organisms. The −1 PRF is prevalent and abundant and the most well-defined −1 PRF phenomena are directed by an mRNA sequence motif composed of the following three crucial elements^11^: 1) the so-called slippery sequence composed of seven nucleotides; 2) a short spacer sequence (usually less than 12 nt); and 3) a downstream stimulatory structure (usually a pseudoknot or a stem-loop). Compared with −1 PRF, +1PRF has fewer examples found in bacteria, fungi, mammals and ciliated protozoa of *Euplotes*. In the majority of bacteria, +1 PRF reportedly regulates the expression of release factor 2 (RF2)^12^,^13^; in fungi and mammals, +1 PRF purportedly regulates the expression of ornithine decarboxylase antizyme (OAZ), the negative regulator of cellular polyamine levels^14^,^15^.

Unlike −1 PRF, which has only one well-understood type of frameshift signal, +1 PRF involves highly diverse mechanisms. In *Escherichia coli*, RF2 autoregulates its production by the in-frame UGA premature termination codon found within the slippery site U CUU UGA. Peptide chain termination is efficient when adequate RF2 is present, thereby suppressing +1 PRF and limiting its translational production. However, low RF2 levels result in the inefficient recognition of the UGA codon and thus increased efficiency of +1 PRF, thereby allowing the expression of the RF2 protein^16^. In addition, a Shine–Dalgarno–like (SD–like) element located upstream of the slippery site can stimulate a +1 frameshift by interacting with the anti–SD sequence on the 16S rRNA^17^. In eukaryotes, +1 PRF is driven by other mechanisms. In the case of human OAZ mRNA, the crucial stimulatory element is the mRNA secondary structure located downstream of the slippery sequence. Similar to RF2 from *E. coli*, the OAZ +1 frameshift is stimulated by a 0-frame UGA codon, and is also autoregulated. Ornithine decarboxylase (ODC) catalyses the first step in polyamine biosynthesis, whereas OAZ downregulates polyamine synthesis by stimulating the ubiquitin-independent degradation of ODC by the proteasome. Thus, the increased levels of polyamines cause a negative feedback on polyamine synthesis by stimulating +1 PRF, and hence OAZ synthesis^18^.

*Euplotes* contains several +1 PRF genes, such as the Tec2 transposon ORF2 protein^19^,^20^, membrane occupation and recognition nexus (MORN) repeat protein, C_2_H_2_-type zinc finger protein, Ser/Thr protein kinase^6^, cAMP-dependent protein kinase^21^, nuclear protein kinase^22^, La motif protein^23^, mitogen-activated protein kinase (MAPK1)^24^,^25^, and the reverse transcriptase subunits of telomerase^26^,^27^,^28^. All of these genes have some common features. Their slippery sequences usually have the motif 5′-AAA-TAR-3′ (where R=A or G, the underlined sequence denotes the 0-frame codons), and all genes require a +1 PRF to produce complete protein products. In addition, a previous survey^6^ of *Euplotes crassus* macronuclear genes by random sequencing has found three new putative +1 PRF genes from 23 macronuclear genes, suggesting that the frequency of genes requiring frameshifts may exceed 10%.

The present study conducted a genome-wide investigation of +1 PRF in *E. octocarinatus* through genome and transcriptome sequencing. A total of 3,700 (approximately 11%) putative +1 PRF genes were identified in *E. octocarinatus.* To the best of our knowledge, this frequency of +1 PRF is the highest found in all living organisms. Based on the functional annotation of Pfam, GO and KEGG, we systematically investigated the putative functions of +1 PRF gene products, which were mainly enriched in protein kinases. We also found a novel suppressor tRNA of UAA which is a potential key factor of +1 PRF in euplotids. This work provides the first comprehensive genome-wide investigation of +1 PRF in *E. octocarinatus*, and thus lays a foundation for further exploring the mechanism of PRF.

## Results and Discussion

### Constructing the transcripts of *E. octocarinatus* by genome and transcriptome sequencing

The PRF occurs at the post-transcriptional level; thus, transcripts should first be assembled to analyse the PRF. Reference-based transcriptome assembly is reported as the best method to construct transcripts, especially full-length transcripts, from short high-throughput sequencing reads^29^. The MAC genome and transcriptome of *E. octocarinatus* were sequenced to construct a high quality transcripts set.

In consideration of the unique properties of the highly fragmented macronuclear genome of *Euplotes*, two short paired-end sequencing libraries with insert sizes of 180 bp (100 bp × 2 by Illumina Hiseq2000 platform) and 500 bp (300 bp × 2 by Illumina Miseq platform) were constructed and sequenced. In total, about 11 gigabases (Gb) were obtained (Table S1). Several popular short-read assemblers were tested and compared to obtain full-length nanochromosomes and the minimum number of contigs simultaneously (Table 1). Finally, we adopted the strategy in Figure S1. In general, HiSeq and Miseq data were independently assembled using the assembler with the best performance. Specifically, the Miseq data (300 bp × 2) were assembled using Mira. This assembly produced a large proportion of 2-telomere contigs (61.0%) and long contigs (N50 length 2,683 bp). However, the Miseq assembly missed many nanochromosomes which were shorter than 500 bp because of the library insert size limitation (Table 1). Therefore, the Hiseq data (100 bp × 2) was independently assembled using SPAdes. This assembly produced 24.8% of 2-telomere capped contigs with a N50 length of 1,129 bp. Then, the two assemblies were merged using CAP3, and all redundant contigs were removed on the basis of the result of LASTZ (see Methods and Fig. S1). However, some telomereless contigs (5,494) shorter than 500 bp were not removed from the final assembly. We speculate that those ‘chaff’ contigs were fragments of the MIC or the inter-genic regions of some long chromosomes with a low GC content (Fig. S2).

**Figure 1:**
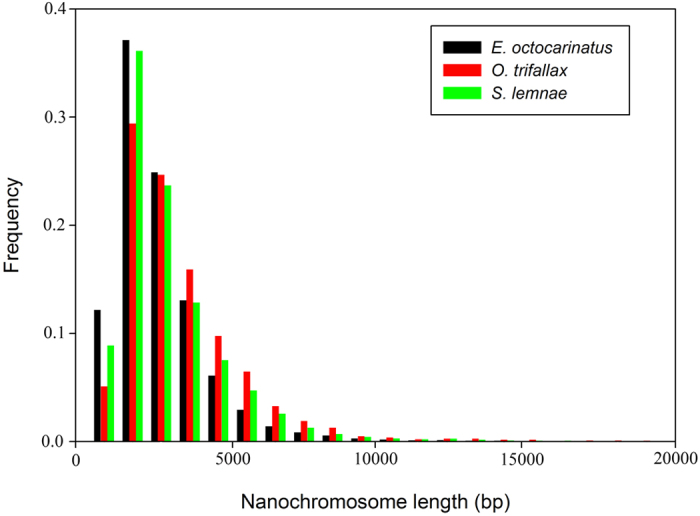
Length distribution of nanochromosomes of three highly fragmented macronuclear genomes. X axis is the contig length (nucleotides), Y axis is the frequency of contigs with the indicated lengths. The histograms show normalized frequencies for 29,413 nanochromosomes of *Euplotes octocarinatus*, 15,085 nanochromosomes of *Oxytricha trifallax* and 16,029 nanochromosomes of *Stylonychia lemnae*.

Based on the GC content results of all contigs (Fig. S3), we suspected that the initial assembly contained a mixture of target DNA, bacterial DNA (endosymbionts of *E. octocarinatus*) and mitochondrial DNA (some sub-peaks were located behind the major peak). Therefore, a series of filters was applied to exclude the contamination (see Methods), and 1,628 bacterial contigs and 72 mitochondrial contigs were removed from the initial assembly. Finally, a total of 41,980 contigs with an average length of 2,117 bp were used as the *E. octocarinatus* MAC genome assembly, and most (70.1%) of these contigs were capped with telomeres on both ends. The completeness of the genome was supported by the assessment results (see Methods). Subsequently, we compared two reported highly fragmented macronuclear genomes^30^,^31^ with the *E. octocarinatus* assembly. Similar to *Oxytricha* and *Stylonychia*, few nanochromosomes were assembled at either extremities of the length distribution in *Euplotes* (Fig. 1). Only 283 were shorter than 500 bp and 15 were longer than 15 kb.

To construct the transcript set, high-throughput RNA-seq (125 bp × 2) of *E. octocarinatus* was performed (see Methods). We obtained 39,478,354 short reads, with a total length of more than 4.9 Gb through sequencing. Low-quality reads were filtered by fastq_quality_filter with the parameters -q 20 -p 80. Then high-quality reads of RNA-seq data were mapped to the *Euplotes* macronuclear genome by Tophat^32^, and all mapped reads were assembled using Cufflinks^33^. Finally, 32,353 transcripts were generated with a mean transcript length of 1,300 bp and a N50 of 1,578 bp.

### High frequency of +1 PRF in *E. octocarinatus*

**Figure 2:**
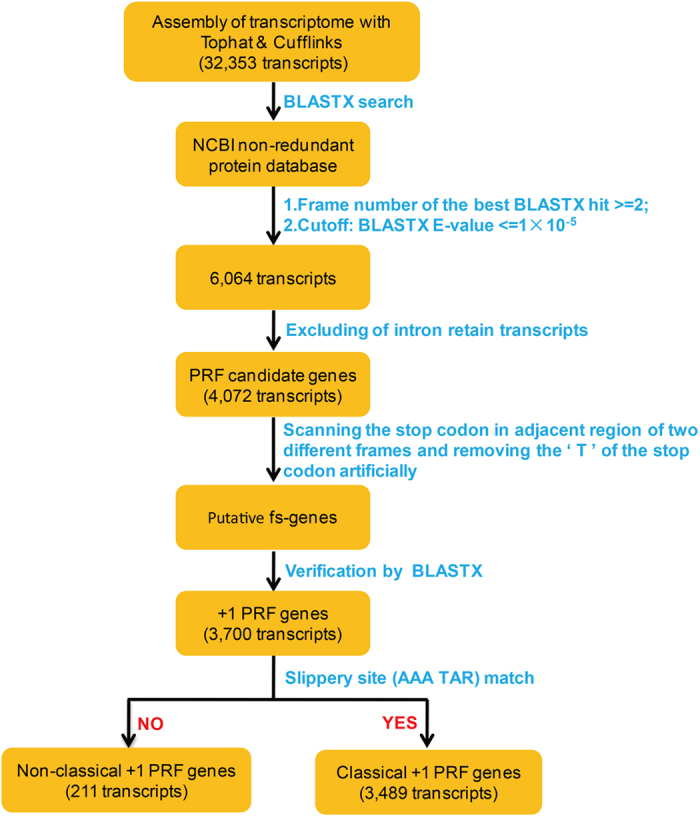
Pipeline of prediction of +1 programmed ribosomal frameshifted transcripts.

A similarity search-based method was used to identify the +1 PRF transcripts in *E. octocarinatus*. The strategy was to find out-of-frame ORFs first and then identify the frameshift motif in the in-frame ORF which could potentially redirect ribosomes from the upstream ORF into the downstream one, resulting in the translation of a complete protein. As depicted in Fig. 2, all transcripts were aligned to the NCBI non-redundant (nr) protein database by using BLASTX with a cut-off of 10^−5^. A total of 6,064 transcripts having two or more high score fragments with different reading frames in the same hit protein sequence were extracted on the basis of BLASTX results. Considering that the intron retention transcripts may also direct the production of out-of-frame ORFs and lead to BLASTX results similar to PRF genes, we identified and excluded intron retention transcripts by using transcriptome information. Based on the typical arrangement of +1 frameshift genes in *Euplotes*, the initial open reading frame is expected to terminate with the sequence 5′-AAA TAR-3′. So the stop codon (TAA or TAG) was searched in the initial open reading frame, and the ‘T’ of the stop codon was artificially removed. Subsequently, this new *fs*-gene was aligned to the NCBI nr protein database by BLASTX again. Once a C-terminally extended protein was produced, this gene would be marked as a +1 PRF gene. Using this strategy, we identified 3,700 putative +1 PRF genes from the 32,353 *E. octocarinatus* transcripts. In addition to the 3,489 +1 PRF genes with the classical ‘*Euplotes* frameshift motif’ (5′-AAA-TAR-3′), we also identified 211 novel +1 PRF genes with different types of slippery sequences. Among these novel +1 PRF genes, the most abundant slippery sequence motif was the 5′-TTT-TAR-3′ motif with 54 genes (Table S2), followed by the 5′-AAG-TAR-3′ motif with 41 genes, the 5′-AAT-TAR-3′ motif with 29 genes, and the 5′-ATT-TAR-3′ motif with 28 genes.

Thus, the present study has increased the number of previously known +1 PRF genes in *E. octocarinatus* by three orders of magnitude. As expected, two previously reported +1 PRF genes in *E. octocarinatus* –cAMP-dependent protein kinase^21^ (CUFF.28794.1) and putative nuclear protein kinases^22^ (CUFF.8279.1) – were identified by our pipeline, suggesting that the method we used was robust. Detailed information on the putative +1 PRF transcripts, including length, GC content, coordinates of predicted slippery sit and E-value of BLASTX is presented in Table S3.

The genome-wide analysis of the *Saccharomyces cerevisiae* genome^11^ and 1,106 complete prokaryotic genomes^34^ suggests a high frequency of −1 PRF in these organisms. However, only a few of the +1 PRF genes have been described (Table 2). By contrast, no −1 PRF gene has been reported in *Euplotes* so far, but several +1 PRF cases have been reported. Our results showed that approximately 11.4% genes required +1 PRF to produce a functional protein in *E. octocarinatus*. Our results provide evidence supporting the notion that euplotids contain an extremely high number of genes requiring +1 frameshifts for expression at the post-transcriptional level. The observed number of +1 PRF genes was higher in *E. octocarinatus* than in other organisms (Table 2), but the true percentage of frameshifted genes in *E. octocarinatus* should be more abundant, because only 52.4% of the transcripts (16,950 of 32,353) have a homologous gene in other organisms. In specific, about half of the transcripts whose functions are unknown may also require a frameshift for expression.

### Hypothetical function of +1 PRF gene products is significantly enriched in protein kinases

We systematically investigated the hypothetical function of 3,700 identified +1 PRF genes. A total of 2,336 putative +1 PRF genes were found to contain at least one protein domain by searching the Pfam database. The most abundant protein domain found in those genes was ‘Pkinase’ domain (PF00069.20), with a total of 362 genes (Table S4), followed by the ‘MORN’ domain (PF02493.15) with 265 genes, the ‘WD40’ domain (PF00400.27) with 179 genes, the ‘SHIPPO-rpt’ domain (PF07004.7) with 146 genes, and the ‘cNMP_binding’ domain (PF00027.24) with 130 genes. All putative +1 PRF genes were mapped to the Kyoto Encyclopedia of Genes and Genomes (KEGG)^35^ pathway to investigate the biological pathways where the putative +1 PRF genes may be involved. In general, 813 genes were assigned to 282 KEGG pathways (Table S5). The pathways represented by the putative +1 PRF genes included the PI3K-Akt signalling pathway (18 members), the sphingolipid signalling pathway (14 members) and the MAPK signalling pathway (12 members). Furthermore, a total of 1,629 putative +1 PRF transcripts were annotated with at least one GO term and categorised into 26 functional groups on the basis of sequence homology (Fig. S4). In each of the three main categories, namely, GO classification cellular component, molecular function and biological process, the terms ‘cell’ and ‘cell part’, ‘binding’ and ‘catalytic’, and ‘cellular process’ were dominant, respectively.

Functional annotations indicated that the putative +1 PRF genes in *E. octocarinatus* possessed various functions involved in multiple cellular processes and pathways. As reported previously, most putative +1 PRF genes encode proteins with enzymatic functions, especially protein kinases^6^. However, none of the highly expressed genes in cells have been reported to require a frameshift which is proven by the fact that the expression abundance of putative +1 PRF genes [mean fragments per kilobase of transcript per million mapped fragments (FPKM) value: 10.59] was significantly lower than that of normal genes (mean FPKM value: 44.38) (*p* < 0.01*, t* test). The most abundant representative proteins in the cell were ribosomal proteins. All 79 of the standard eukaryotic ribosomal proteins of *E. octocarinatus* (32 small subunit and 47 large subunit proteins) were identified and analysed, and none of them required a frameshift for expression.

**Figure 3:**
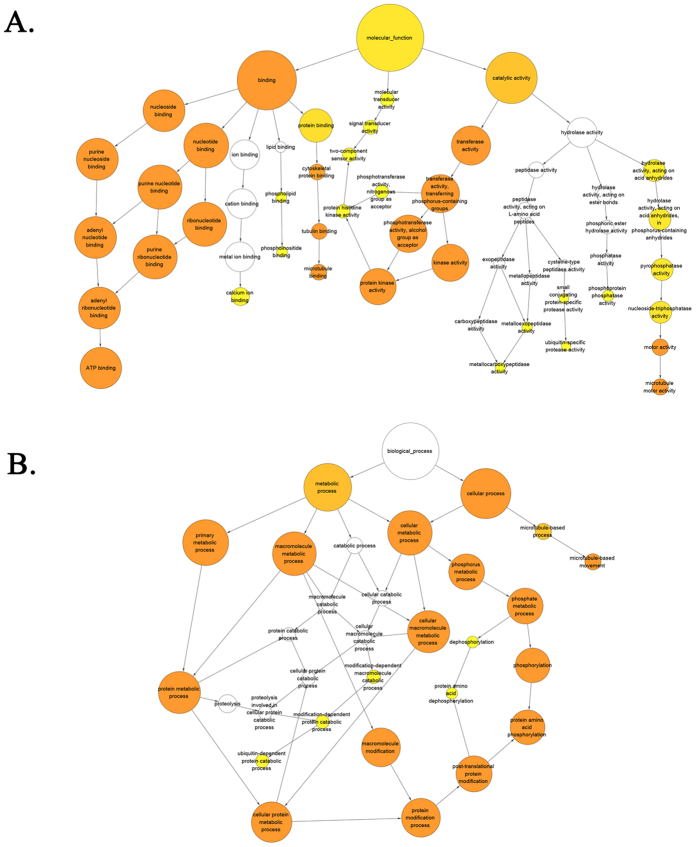
Enriched GO terms of +1 PRF transcripts were analyzed with Bingo. Each circle represents a GO term, arrows indicate pairs of GO terms with a parent-child relationship. Colored circles are statistically significant overrepresented GO terms (functions); the deeper of the color, the smaller the corrected p-value (more significant). Overrepresented molecular functions (**A**) and biological processes (**B**).

A GO enrichment analysis of putative +1 PRF genes was performed to investigate the functional enrichment of putative +1 PRF genes. Results showed that the identified putative +1 PRF genes were significantly overrepresented in the regulation of various biological processes such as dephosphorylation, protein amino acid phosphorylation, and ubiquitin-dependent protein catabolic process (Fig. 3).

These results suggest that the products of these putative +1 PRF genes in *E. octocarinatus* are significantly enriched in protein kinases. Protein kinases are important regulatory components of every eukaryotic intracellular signal transduction pathway. Some protein kinases, such as MAPK1, are associated with cell proliferation and cell cycle events; MAPK1 is a homologous kinase with intestinal–cell kinases in mammals. The expression of the *MAPK1* gene requires +1 translational frameshifting in both *Euplotes raikovi*^24^ and *Euplotes nobilii*^25^. This *Euplotes* kinase is related to the autocrine signalling loop that promotes vegetative growth. Furthermore, the MAPK1 of *E. raikovi* resides in the nuclear apparatus, where it appears either phosphorylated in growing cells which interact in autocrine fashion with their own specific (self) signalling pheromones or dephosphorylated in cells which are induced to mate and temporarily arrest their growth by paracrine interactions with foreign (non-self) signalling pheromones^24^. These results suggest that +1 PRF genes may have important functions in cell growth.

### Suppressor tRNA may play an important role in +1 frameshifting in *E. octocarinatus*

**Figure 4:**
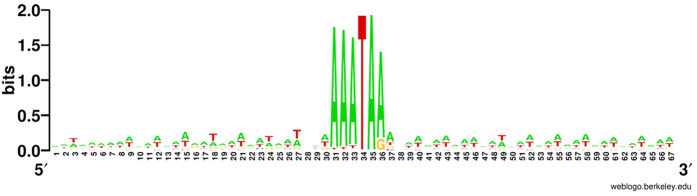
WebLogo displaying conserved sequences associated with frameshift sites. Sizes of letters denote information content, or sequence conservation, at each position. The analysis is based on the alignment of 30 bp preceding and following the frameshift motif from 4,545 predicted slippery sites.

With sufficient samples of +1 PRF genes, the conserved sequence elements which might facilitate frameshifting were checked. To search the potential conserved sequence elements, 30 bp upstream and downstream of the conserved slippery sequence motif from 4,545 predicted slippery sites were extracted and analysed using WebLogo^36^. Consistent with a previous report, no conserved sequence element was found except the slippery site sequence 5′-WWW TAR-3′ (W=A or T, Fig. 4), suggesting that frameshifting does not depend on other sequence motifs.

**Figure 5:**
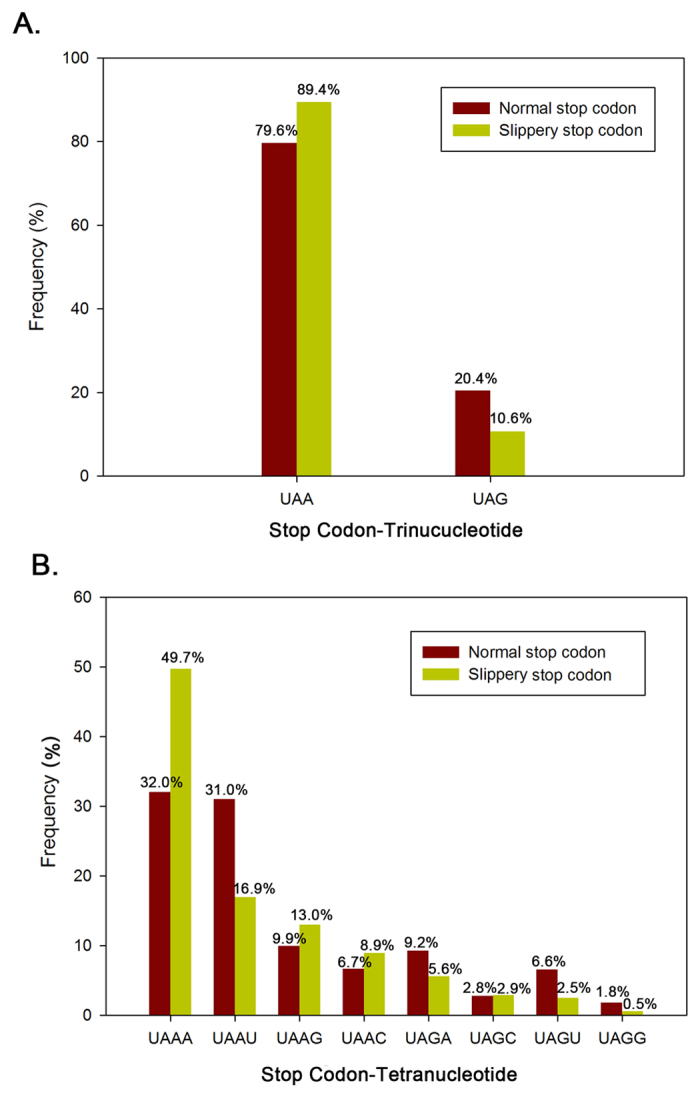
Frequency of stop codon usage in *Euplotes octocarinatus.* (**A**) Frequency of stop codon-trinucucleotide. (**B**) Frequency of stop codon-tetranucleotide. The red and yellow bars indicate normal termination signal and slippery signal, respectively.

Klobutcher and Farabaugh^37^ suggested that altering eRF1 to ignore UGA might impair its recognition of other termination codons in *Eupotes*. In addition, Vallabhaneni *et al.*^38^ proved that the reassignment of UGA to Cys in *E. octocarinatus* increases +1 slippery stop frameshifting at both UAA and UAG. Based on this finding, we analysed the stop codon usage in *E. octocarinatus* at the transcriptome level, and compared the usage between the ‘normal’ stop codon and the slippery stop codon. Results showed that UAA was preferentially used in both the ‘normal’ termination signal (79.6%) and the slippery signal (89.4%) (Fig. 5A). Moreover, the frequency of UAA codon usage in slippery signal is significantly higher than that in ‘normal’ termination signal (P < 0.01, Fisher exact test) which suggested that UAA may be favourable for frameshifting in *E. octocarinatus*. The release factor recognises a tetranucleotide sequence consisting of the termination codon and its nearest 3′ neighbour nucleotide in both prokaryotes and eukaryotes^37^. Thus, we also analysed the frequency of the tetranucleotide sequence in both normal and +1 PRF slippery sites (Fig. 5B). A similar trend was found in both cases, where UAA-A was the most frequently used tetranucleotide (49.7% in the slippery signal and 32.0% in the ‘normal’ termination signal) and UAG-G was the least frequently used (0.5% in the slippery signal and 1.8% in the ‘normal’ termination signal). However, the usage frequency of UAA-A (49.7%) was considerable higher than that of UAA-U (16.9%) in the slippery signals, even though they have similar frequencies in the “normal” termination signals (32.0% vs. 31.0%). These results suggest that the tetranucleotide sequence UAA-A may be favourable for frameshifting in *E. octocarinatus.*

**Figure 6:**
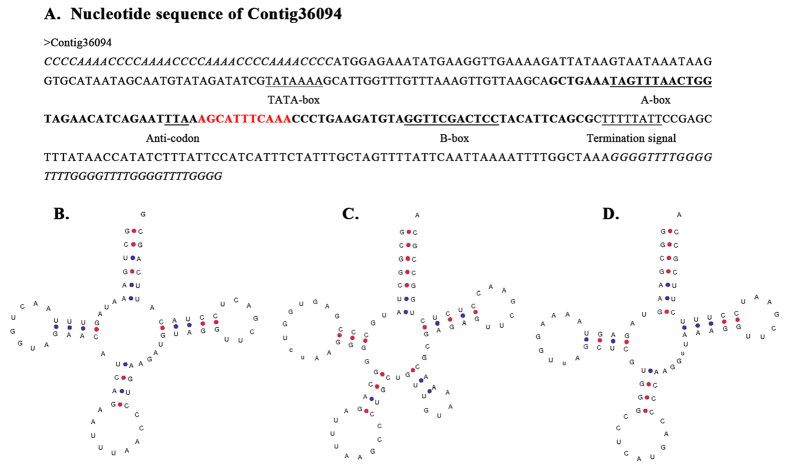
Nucleotide sequences of novel tRNA from different eukaryotic organisms shown in the cloverleaf arrangement. (**A**) The nucleotide sequence of Contig36094 is shown. The coding region is indicated by bold face and the terminal C_4_A_4_ and G_4_T_4_ telomeric sequences are shown in italic. The intragenic promoter boxes, the termination signal and a putative binding for the TATA-box binding protein are underlined. The putative intron is shown in red. The predicted cloverleaf structures of three novel tRNAs from (**B**) *Euplotes octocarinatus* (**C**) *Schizosaccharomyces pombe* and (**D**) *Astasia longa* are shown.

While no suppressor tRNA of UAA had been previously reported in *Euplotes*, a putative suppressor tRNA of UAA (Contig36094) was predicted from the genomic sequences of *E. octocarinatus*. Contig36094 (343 bp) was predicted to encode a 72 nucleotide tRNA that can fold into the characteristic cloverleaf secondary structure (Fig. 6B). An intervening sequence of 11 base pairs located at the canonical 37/38 position^39^ was also predicted in this gene (Fig. 6A). The gene contains the characteristic internal split promoter and a typical termination signal^40^ (Fig. 6A). Furthermore, a perfect consensus sequence, ‘TATAAAA’, for the TATA-binding protein (TBP) was located at position −35 to −29 relative to the +1 nt of the tRNA^UAA^.

Further analysis indicated a base mismatch in the anticodon stem of the molecule, which increased the loop of unpaired bases from the typical seven to nine (Fig. 6B). Such an unusual structure was not unprecedented, and two examples of apparently nine-base anticodon loops in presumably wild-type, functional tRNAs were observed, namely, a tRNA^Leu^ in *Schizosaccharomyces pombe*^41^ (Fig. 6C) and a tRNA^Met^ in *Astasia longa*^42^ (Fig. 6D). In addition, the similar suppressor tRNAs that have been isolated from both bacteria^43^,^44^,^45^,^46^ and yeast^47^,^48^, contained additional nucleotides in their anticodon loops. Expanded or modification-deficient anticodon stem loops have been proven to cause the ribosome to decode four rather than three nucleotides, resulting in a +1 translational frameshifting^49^,^50^,^51^. Therefore, we proposed that the particular suppressor tRNA^UAA^ enters the ribosomal A site and decode 4 nucleotides when the translating ribosome meets the slippery stop codon. Then translation would continue in the +1 frame. Further experimental verification is needed to investigate how suppressor tRNA^UAA^ regulates +1 PRF in *E. octocarinatus*.

## Conclusions

We reported a genome-wide investigation of +1 PRF in *E. octocarinatus* on the basis of its genome and transcriptome sequencing. We identified 3,700 (about 11%) putative +1 PRF genes, which to the best of our knowledge, is the highest frequency of +1 PRF found in all living organisms up to date. We also found a novel suppressor tRNA of UAA, which is potentially the key factor of +1 PRF in euplotids. This work provided the first comprehensive genome-wide investigation of +1 PRF and contributed to the mechanism of underlying programmed translational frameshift.

## Methods

### Cell culture, DNA isolation, and genome sequencing

*E. octocarinatus* line 69 was cultured in 2 liter flasks containing in synthetic medium^52^ at room temperature with the photosynthetic flagellate *Chlorogonium elongatum* as a food source. This strain was kindly provided by Klaus Heckmann (Universität Münster, Germany). Prior to harvesting, *Euplotes* cells were starved for 7–10 days to allow them to exhaust most of the food. Then, 8–10 liters of starved cells were harvested by filtering through several layers of gauze to remove large particles, and then a filter paper was used to concentrate cells and remove bacteria and small contaminants. Cells were collected by centrifugation (4 °C,4,000 rpm, 5 min) and then lysed in Urea buffer (0.01 M Tris-HCl, 0.01 M EDTA, 0.35 M NaCl, 1% SDS, 42% Urea, pH 7.4) for 5 min at 4 °C. After phenol/chloroform extraction, total DNA was dialyzed against isopropanol followed by ethanol precipitation. RNase was then added and incubated for 1hr at 37 °C.

According to the whole genome shotgun strategy, genomic DNA was broken into random fragments. Two libraries with different paired-end (PE) length distributions were created from *Euplotes* DNA sequence data (Table S1). The 500 bp library was sequenced using the Illumina MiSeq platform, and the 180 bp library was sequenced using the Illumina HiSeq 2000 platform.

### The genome assembly and assembly cleanup

The genome was assembled by a meta-assembly method (Fig. S1). All sequence data were used to build a reference genome.

MiSeq reads were assembled with Mira (4.0)^53^. High quality reads were selected for assembly. Read1 and Read2 were trimmed by fastx_trimmer (from the FASTX-Toolkit) with the parameters -l 290 and -l 250, respectively, and then filtered by fastq_quality_filter (from the FASTX-Toolkit) with the parameters -q 20 -p 80. FLASH (1.2.10)^54^ was used to merge these processed paired-end reads with the parameters –M 100. Finally, approximately 3.5 Gb reads were assembled with Mira (default parameters). SPAdes (2.5.0)^55^ was run with the “careful” option on HiSeq reads. Then we merged two assembly results with the CAP3 assembler with strict overlap parameters (-o 50 -p 99).

To remove redundant contigs from the assembly, LASTZ^56^ was used to align every contig to each other. Contigs were discarded (13,623 in total) if they had longer non-self matches that are identical or almost identical (≥90% coverage and ≥90% sequence identical) (Fig. S1).

The final assembly contained a mixture of bacterial DNA and mitochondrial DNA. To identify bacterial genomic sequences, all telomereless contigs were searched against NCBI non-redundant protein sequences database using BLASTX (E-value ≤ 1e^−5^). Any contig that belongs to bacteria or archaea was removed. To exclude mitochondrial contamination in our final assembly, these telomereless contigs which had substantial TBLASTX matches (E-value ≤ 1e^−4^) to the *Euplotes minuta and Euplotes crassus* mitochondrial genome^57^ were removed. A total of 1628 bacterial contigs and 72 mitochondrial contigs were identified and removed. In addition, we also removed 35 contigs that were shorter than 100 bp.

### Assessment of genome completeness

To assess the completeness of the draft *Euplotes* macronuclear genome, we used a strategy similar to that used to assess completeness of *Oxytricha*^30^.

Firstly, we evaluated the percentage of reads mapping to the final assembly. All reads and reads containing telomere sequences of HiSeq reads were separately mapped to the final assembly with BWA^58^ (default parameters; version 0.7.5). Nearly all high-quality reads mapped to our final assembly (96% of all PE reads and 92% of telomeric reads). Furthermore, the majority of contigs (70.1%) had both 5′ and 3′ telomeres (Table 1). This simple assessment indicated that our assembly was largely complete.

Then we analyzed the completeness of two gene sets: ribosomal proteins and tRNAs. Based on the reciprocal blast results, all 80 of the standard eukaryotic ribosomal proteins except L41 were identified (32 small subunit and 47 large subunit proteins). Considering that the coding sequence of human L41 is too short (only 75 bp), we speculated that the L41of *Euplotes* probably was missed in the process of library construction. In addition, tRNAscan (version 1.3.1)^59^ with default parameters was used to search for tRNAs. A total of 95 contigs of *Euplotes’s* macronuclear genome encode a comprehensive set of tRNAs for all 20 standard amino acids (Table S6) including a novel suppressor tRNAs of traditional stop codon UAA.

We also assessed the completeness of the macronuclear genome by searching protein sequences from *Euplotes* against the core eukaryotic genes (CEGs)^60^. Matches from BLASTP with E-values lower than 1e-10 and a sequence coverage ≥70% of the CEG sequence were counted as a match. Of the predicted proteins, 218 proteins were predicted for *Euplotes* and had substantial sequence similarity to the CEG protein sequences. 21 of the 30 remaining CEGs were found by TBLASTN matches or using HMMER3^61^ domain searches because of the deep evolutionary divergences of ciliates from these eukaryotes. After these more sensitive searches in *Euplotes,* only 6 CEGs are missing from the 245 ciliate-specific CEGs. Of the six undetectable ciliate-specific CEGs, one, KOG3285, is also missing from *Oxytricha*^30^ and *Stylonychia*^31^. Thus, the macronuclear genomes of *Euplotes* encode 97.6% of the ciliate-specific CEGs.

### RNA isolation and transcriptome sequencing

Cell culture and collection were the same as described for DNA isolation. Total RNA was extracted using the RNeasy Plus Mini Kit Cell Mini Kit (Qiagen) per manufacturer’s instructions. Total RNA concentrations was determined using Qubit RNA Assay Kit in Qubit 2.0 Flurometer (Life Technologies, CA, USA) and RNA integrity was assessed using the RNA Nano 6000 Assay Kit of the Agilent Bioanalyzer 2100 system (Agilent Technologies,CA, USA).

Poly-A mRNAs was purified using Dynal magnetic beads (Invitrogen). Double-stranded cDNAs were synthesized using reverse transcriptase and random hexamer primers. cDNAs were fragmented by nebulization, and the standard Illumina protocol was followed thereafter to construct mRNA-seq libraries. The normalized cDNA population was sequenced using the Illumina 2000 platform, with paired-end 125 bp mode. About 4.9 Gb of raw RNA-seq data were obtained.

### Gene prediction

Given that the existence of PRF genes will influence the accuracy of gene prediction, all +1 PRF candidate genes were excluded. The *de novo* prediction software AUGUSTUS (version 3.0.2)^62^ was used to predict complete genes on the non-PRF contigs (38,615 in total).

To obtain a reliable training data set, all non-PRF transcripts of Cufflinks^33^ assembly were aligned to the *Euplotes* macronuclear genome and reassembled by PASA (version r20140417, run with default parameters)^63^. These transcripts which were marked as “complete” (had both ATG and TAA/TAG) were searched against NCBI non-redundant protein sequences database using BLASTX. Matches from BLASTX with E-values lower than 1e-10 and a sequence coverage ≥95% of the top hit sequence were extracted. In addition, we only chose proteins that were < 70% identical to each other according to the recommendations in the AUGUSTUS documentation. Ultimately, 551 *Euplotes* genes were used to train AUGUSTUS. The final data set of 551 genes was split into training and test data sets of 401 and 150 genes respectively. For gene prediction, AUGUSTUS was run with the following parameters: “–species=euplotes –UTR=on –extrinsicCfgFile=install/augustus.3.0.2/config/extrinsic/extrinsic.M.RM.E.W.cfg –genemodel=complete –codingseq=on”. To produce additional constraints (hints) for AUGUSTUS, the RNA-seq data were processed according to the instructions on the website (http://www.molecularevolution.org/molevolfiles/exercises/augustus/prediction.html#prephints). We also recompiled AUGUSTUS after decreasing the default minimum length of intron hints in the source code from 39 to 25 bp to allow AUGUSTUS to evaluate hints for shorter introns^30^.

Overall, 29,076 putative protein-coding genes were obtained, and 90% of them were supported by RNA-Seq Reads. About 89% nanochromosomes were predicted containing one or more complete gene. Key properties of *Euplotes*’s gene predictions are given in Table S7. Like other ciliates, the noncoding regions of *Euplotes* are more AT-richer than coding regions (e.g., 23.3% GC content in introns, versus 31.3% GC content in exons).

### Functional annotation and enrichment analysis

The ‘T’ of the stop codon within the slippery sequence was artificially removed. Then these new *fs*-genes and non-PRF transcripts were translated into amino acid sequences using the GetOrf program in the EMBOSS package^64^ with the *Euplotid* Nuclear Code, and the longest CDSs were obtained by using a custom Perl script. Subsequently, the amino acid sequences were loaded into Pfamscan^65^ to perform protein domain annotation, and only Pfam-A database was searched.

Gene ontology (GO) annotations were performed by mapping of GO terms to Pfam entries. This mapping was generated from data supplied by InterPro^66^ for the InterPro2GO mapping. Functional enrichment analysis of +1 PRF genes was performed to determine the significantly enriched GO terms and relevant proteins by using BINGO^67^ plugin in the Cytoscape platform (version 3.1.1)^68^. Enrichment analysis of GO term assignment was performed in reference to the entire *E. octocarinatus* transcripts (containing 7,060 proteins). The corrected (corr) p-values were derived from a hypergeometric test followed by Benjamini and Hochberg false discovery rate (FDR) correction. The FDR ≤ 0.05 was regarded as significant.

In addition, the +1 PRF transcripts were annotated according to the Kyoto Encyclopedia of Genes and Genomes (KEGG)^35^ orthology (KO) using the KEGG Automatic Annotation Server (KAAS)^69^. The amino acid sequences of translated +1 PRF transcripts were used as the query sequence, and the bi-directional best hit (BBH) method was employed to obtain the KO terms for the query sequences.

### Availability of supporting data

The BioProject accession number for the genome is PRJNA294366. The raw genome sequences reads have been deposited in Sequence Read Archive (SRA) under accession SRX1267944 and SRX1270715. Transcriptome data has also been deposited in SRA under accession SRX1270740.

## Additional Information

**How to cite this article**: Wang, R. *et al.* High frequency of +1 programmed ribosomal frameshifting in *Euplotes octocarinatus*. *Sci. Rep.* **6**, 21139; doi: 10.1038/srep21139 (2016).

## Supplementary Material

Supplementary Information

Supplementary TableS3

## Figures and Tables

**Table 1:** Comparison of *Euplotes octocarinatus* macronuclear genome assemblies.

Assembler	ABySS	SOAP	SPAdes	Mira	SPAdes	Final Assembly
Assembler version	1.5.1	2.04	2.5.0	4.0.2	2.5.0	–
Data	HiSeq Data	HiSeq Data	HiSeq Data	MiSeq Data	MiSeq Data	–
Assembly size (Mb)	83.0	97.8	84.6	126.2	107.2	88.9
Contigs (n)	241,146	180,079	74,880	56,612	68,352	41,980
N50 (bp)	1,144	1,650	2,034	2,683	2,443	2,947
Mean contig length (bp)	344	543	1129	2228	1,619	2117
Max contig length (bp)	18,818	17,403	18,769	53,269	195,456	53,269
Number of 2-telomere contigs (n)	13,935	6676	18,556	34,536	27,802	29,413
Number of 1-telomere contigs (n)	32,125	23,449	17,959	12,751	7,548	4,367
Number of 0-telomere contigs (n)	195,085	149,942	38,364	8,749	32,594	7,554
Number of multi-telomere contigs	1	12	1	576	408	645
Number of shorter nanochromosomes (length≤500 bp)	269	137	287	5	68	287
2-telomere contig percentage (%)	5.8	3.7	24.8	61.0	40.7	70.1
Total PE read coverage (%)	82.1	95.9	98.3	84.7	90.2	95.9
Telomeric PE read coverage (%)	72.6	52.1	99.6	88.5	89.7	91.9

**Table 2:** Summary of +1 programmed ribosomal frameshifted genes in diverse organisms.

Organism	Evolutionary Group	Total gene number^a^	+1 PRF gene number^b^	Method & Reference
*Homo sapiens*	Eukaryote (Metazoa)	20,403	3	Experimental verification^70^,^71^,^72^
*Rattus norvegicus*	Eukaryote (Metazoa)	23,636	1	Experimental verification^73^
*Mus musculus*	Eukaryote (Metazoa)	22,556	3	Experimental verification^71^,^72^,^74^
*Xenopus laevis*	Eukaryote (Metazoa)	11,078	1	Experimental verification^75^
*Danio rerio*	Eukaryote (Metazoa)	26,719	2	Experimental verification^76^
*Drosophila melanogaster*	Eukaryote (Metazoa)	13,954	1	Experimental verification^77^
*Caenorhabditis elegans*	Eukaryote (Metazoa)	20,360	1	Experimental verification^75^
*Saccharomyces cerevisiae*	Eukaryote (Fungi)	5,907	3	Experimental verification^18^,^78^,^79^
*Euplotes octocarinatus*	Eukaryote (Ciliate)	~32,353	3,700	Computational analysis This work
*Escherichia coli*	Prokaryote	4,140	1	Experimental verification^12^
*Listeria monocytogenes phage PSA*	Virus	–	2	Experimental verification^80^
*Influenza A virus*	Virus	–	1	Experimental verification^81^

^a^Gene number retrieved from the KEGG genome statistics.

^b^Only non-transposon, protein-coding genes.
