# Supplementary material for: High frequency of +1 programmed ribosomal frameshifting in Euplotes octocarinatus
Source: Sci Rep. 2016 Feb 19;6:21139. doi: 10.1038/srep21139 (PMC4759687; doi:10.1038/srep21139)
Supplement: Supplementary Information [file srep21139-s1.pdf]

High frequency of +1 programmed ribosomal frameshifting in *Euplotes octocarinatus*

Ruanlin Wang[1], Jie Xiong[2], Wei Wang[1], Wei Miao[2] and Aihua Liang[1*]

[1]Key Laboratory of Chemical Biology and Molecular Engineering of Ministry of Education, Institute of Biotechnology, Shanxi University, Taiyuan 030006, China.

[2]Key Laboratory of Aquatic Biodiversity and Conservation, Institute of Hydrobiology, Chinese Academy of Sciences, Wuhan 430072, China.

[*]Corresponding author: Aihua Liang

e-mail: aliang@sxu.edu.cn.

Tel.: +86-351-7018731

Fax.: +86-351-7011499

Running title: programmed ribosomal frameshifting in *Euplotes octocarinatus*

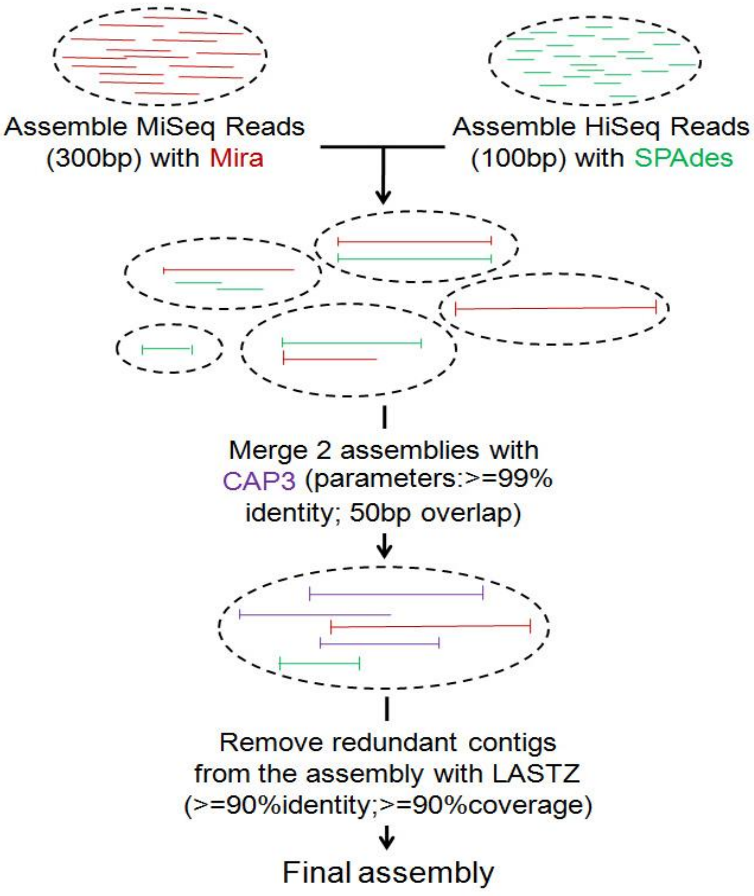

Figure S1. Genome assembly pipeline.

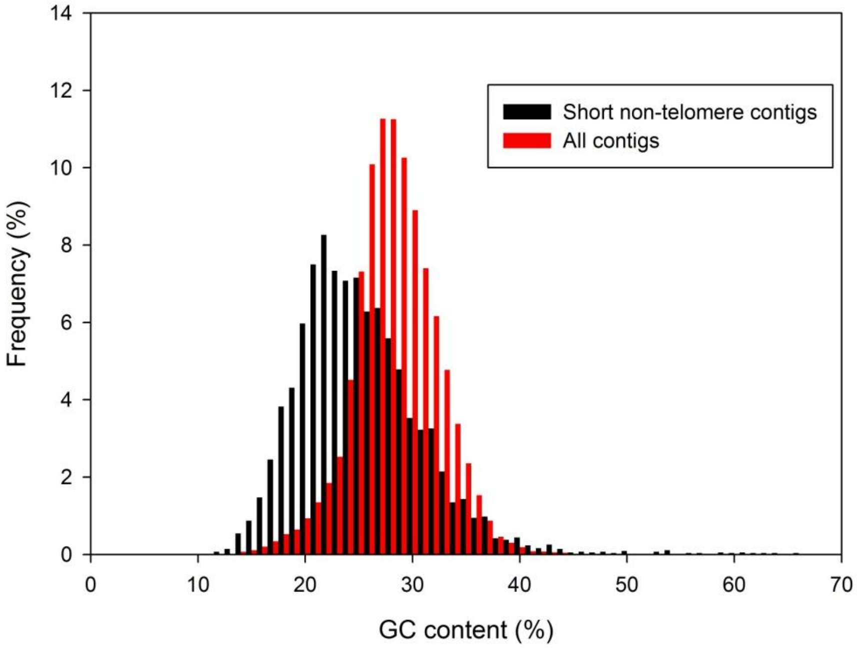

Figure S2. GC content of redundant "chaff" contigs. The black and red bars indicate non-telomere contigs (shorter than 500 bp, 5,494 in total) and all contigs (41,980 in total), respectively.

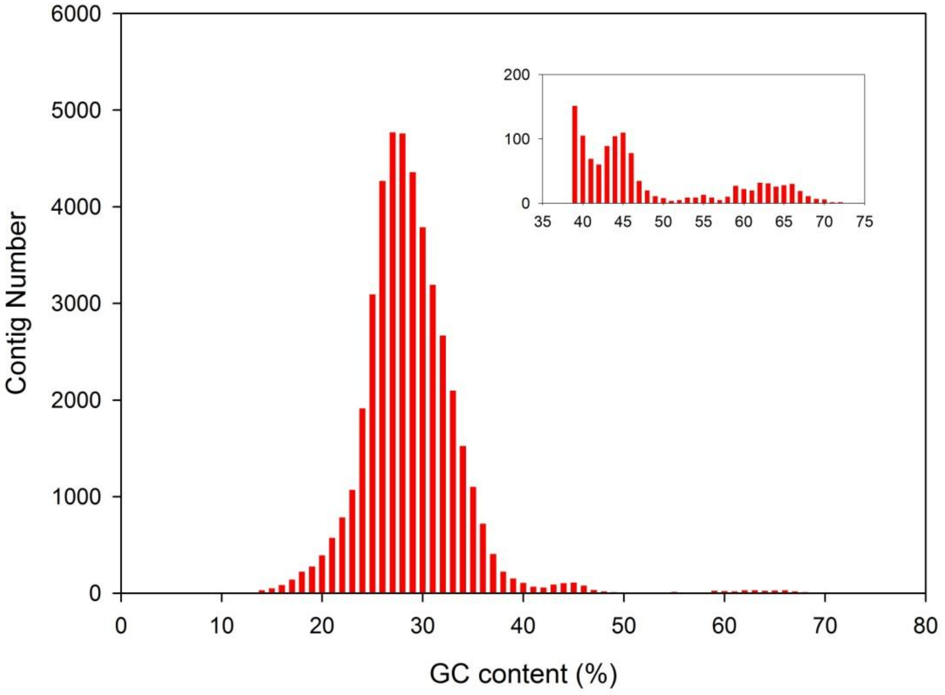

Figure S3. GC content of all contigs. Percentage of GC content is plotted against the number of contigs to show that some sub-peaks are located behind the major peak.

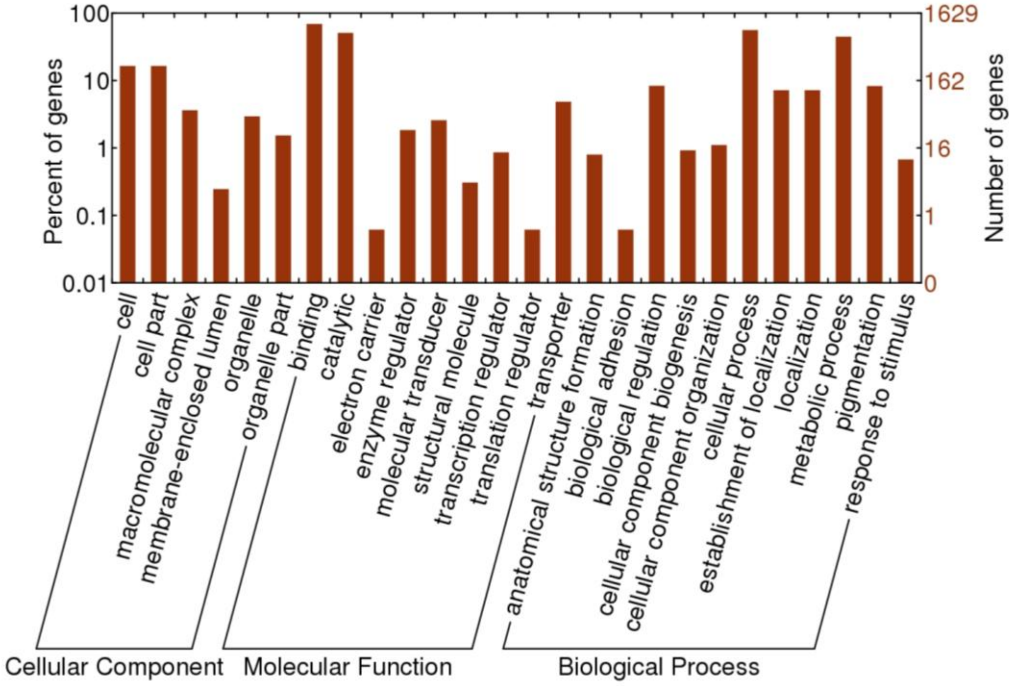

Figure S4. GO categories of the PRF transcripts. The results are summarized in three main categories: biological process, cellular component and molecular function. The y-axis on the right indicates the number of genes in a category. The y-axis on the left indicates the percentage of a specific category of genes in that main category.

Table S1. Parameters of genome sequencing of *Euplotes octocarinatus*

Property	Sequencing Platform	Insert size (bp)	Read Length (bp)	Total Sequences	%GC	Data Size (Gb)
MiSeq reads	Illumina MiSeq	500	300×2	28,684,752	31	8.58
HiSeq reads	Illumina HiSeq 2000	180	100×2	26,811,500	29	2.68
**Total**						~ 11Gb

Table S2. Statistic of novel slippery sequences.

Slippery sequence	Occurrence	Percentage (%)
TTTTAR	54	24.22
AAGTAR	41	18.39
AATTAR	29	13.00
ATTTAR	28	12.56
ACCTAR	10	4.48
AACTAR	6	2.69
CTTTAR	5	2.24
TCATAA	4	1.79
GAATAR	3	1.35
GGGTAA	3	1.35
ACATAR	2	0.90
ACTTAA	2	0.90
AGATAR	2	0.90
AGCTAA	2	0.90
CAATAA	2	0.90
GATTAA	2	0.90
GCCTAR	2	0.90
GTTTAG	2	0.90
TATTAR	3	1.35
TGATAA	2	0.90
TGGTAG	2	0.90
TTCTAA	2	0.90
AGGTAA	1	0.45
ATGTAA	1	0.45
CCATAA	1	0.45
CGATAA	1	0.45
CGTTAA	1	0.45
GAATAA	1	0.45
GGCTAG	1	0.45
GTATAG	1	0.45
TCCTAA	1	0.45
TGCTAA	1	0.45
TTATAA	1	0.45
TTGTAA	1	0.45
ATATAA	1	0.45
ATCTAA	1	0.45
TTATAA	1	0.45

Table S4.The Pfam domain search of PRF transcripts

Domain accession	Domain name	Occurrence
PF00069.20	Pkinase	362
PF02493.15	MORN	265
PF00400.27	WD40	179
PF07004.7	SHIPPO-rpt	146
PF00027.24	cNMP_binding	130
PF13499.1	EF-hand_7	91
PF13948.1	DUF4215	72
PF13516.1	LRR_6	64
PF03133.10	TTL	62
PF00415.13	RCC1	62
PF00225.18	Kinesin	57
PF12796.2	Ank_2	48
PF00520.26	Ion_trans	46
PF02518.21	HATPase_c	44
PF00512.20	HisKA	38
PF01344.20	Kelch_1	36
PF00071.17	Ras	33
PF13414.1	TPR_11	29
PF00806.14	PUF	29
PF00481.16	PP2C	28
PF00612.22	IQ	28
PF01529.15	zf-DHHC	27
PF00443.24	UCH	26
PF00566.13	RabGAP-TBC	26
PF00498.21	FHA	26
PF00515.23	TPR_1	25
PF00782.15	DSPc	25
PF00643.19	zf-B_box	24
PF13833.1	EF-hand_8	24
PF13415.1	Kelch_3	23
PF00005.22	ABC_tran	23
PF13418.1	Kelch_4	22
PF12906.2	RINGv	20
PF00153.22	Mito_carr	20
PF00787.19	PX	19
PF13405.1	EF-hand_6	19
PF08238.7	Sel1	18
PF00246.19	Peptidase_M14	18
PF07690.11	MFS_1	18
PF00664.18	ABC_membrane	18
PF00096.21	zf-C2H2	17
PF00271.26	Helicase_C	17
PF03031.13	NIF	16
PF01485.16	IBR	16

PF00168.25	C2	16
PF12770.2	CHAT	15
PF13879.1	KIAA1430	14
PF13639.1	zf-RING_2	13
PF06337.7	DUSP	13
PF00226.26	DnaJ	13
PF07885.11	Ion_trans_2	12
PF00122.15	E1-E2_ATPase	11
PF00091.20	Tubulin	10
PF13424.1	TPR_12	10
PF00083.19	Sugar_tr	10
PF08016.7	PKD_channel	10
PF00169.24	PH	10
PF00768.15	Peptidase_S11	10
PF00648.16	Peptidase_C2	10
PF00931.17	NB-ARC	10
PF00249.26	Myb_DNA-binding	10
PF13921.1	Myb_DNA-bind_6	10
PF14580.1	LRR_9	10
PF12799.2	LRR_4	10
PF02141.16	DENN	10
PF00004.24	AAA	10
PF07719.12	TPR_2	9
PF00076.17	RRM_1	9
PF13855.1	LRR_8	9
PF02263.14	GBP	9
PF13637.1	Ank_4	9
PF01490.13	Aa_trans	9
PF13913.1	zf-C2HC_2	8
PF01504.13	PIP5K	8
PF00454.22	PI3_PI4_kinase	8
PF00702.21	Hydrolase	8
PF00211.15	Guanylate_cyc	8
PF00270.24	DEAD	8
PF00650.15	CRAL_TRIO	8
PF12695.2	Abhydrolase_5	8
PF07728.9	AAA_5	8
PF02176.13	zf-TRAF	7
PF00097.20	zf-C3HC4	7
PF13181.1	TPR_8	7
PF07534.11	TLD	7
PF01926.18	MMR_HSR1	7
PF00149.23	Metallophos	7
PF01661.16	Macro	7
PF04408.18	HA2	7
PF02841.9	GBP_C	7

PF00036.27	EF-hand_1	7
PF13863.1	DUF4200	7
PF07002.11	Copine	7
PF01067.17	Calpain_III	7
PF00023.25	Ank	7
PF00022.14	Actin	7
PF00098.18	zf-CCHC	6
PF01496.14	V_ATPase_I	6
PF03953.12	Tubulin_C	6
PF13540.1	RCC1_2	6
PF00659.13	POLO_box	6
PF01237.13	Oxysterol_BP	6
PF07717.11	OB_NTP_bind	6
PF01909.18	NTP_transf_2	6
PF12777.2	MT	6
PF13854.1	Kelch_5	6
PF03372.18	Exo_endo_phos	6
PF05708.7	DUF830	6
PF05057.9	DUF676	6
PF03142.10	Chitin_synth_2	6
PF00439.20	Bromodomain	6
PF07707.10	BACK	6
PF01603.15	B56	6
PF07859.8	Abhydrolase_3	6
PF12774.2	AAA_6	6
PF13465.1	zf-H2C2_2	5
PF13920.1	zf-C3HC4_3	5
PF04564.10	U-box	5
PF01167.13	Tub	5
PF01131.15	Topoisom_bac	5
PF01833.19	TIG	5
PF00072.19	Response_reg	5
PF05193.16	Peptidase_M16_C	5
PF00233.14	PDEase_I	5
PF00892.15	EamA	5
PF08393.8	DHC_N2	5
PF00134.18	Cyclin_N	5
PF03556.10	Cullin_binding	5
PF00888.17	Cullin	5
PF03493.13	BK_channel_a	5
PF03747.9	ADP_ribosyl_GH	5
PF12781.2	AAA_9	5
PF12775.2	AAA_7	5
PF13401.1	AAA_22	5
PF00642.19	zf-CCCH	4
PF13894.1	zf-C2H2_4	4

PF13768.1	VWA_3	4
PF01505.13	Vault	4
PF01751.17	Toprim	4
PF03638.10	TCR	4
PF00176.18	SNF2_N	4
PF00160.16	Pro_isomerase	4
PF00613.15	PI3Ka	4
PF03416.14	Peptidase_C54	4
PF04928.12	PAP_central	4
PF08030.7	NAD_binding_6	4
PF05729.7	NACHT	4
PF00560.28	LRR_1	4
PF13246.1	Hydrolase_like2	4
PF03451.9	HELP	4
PF12710.2	HAD	4
PF08022.7	FAD_binding_8	4
PF13202.1	EF-hand_5	4
PF03028.10	Dynein_heavy	4
PF14825.1	DUF4483	4
PF11822.3	DUF3342	4
PF06294.6	DUF1042	4
PF08613.6	Cyclin	4
PF01556.13	CTDII	4
PF04053.9	Coatomer_WDAD	4
PF12348.3	CLASP_N	4
PF00689.16	Cation_ATPase_C	4
PF02214.17	BTB_2	4
PF00651.26	BTB	4
PF02138.13	Beach	4
PF00025.16	Arf	4
PF04547.7	Anoctamin	4
PF00155.16	Aminotran_1_2	4
PF00107.21	ADH_zinc_N	4
PF08240.7	ADH_N	4
PF12780.2	AAA_8	4
PF00300.17	His_Phos_1	3
PF10257.4	RAI16-like	3
PF00102.22	Y_phosphatase	3
PF00654.15	Voltage_CLC	3
PF02151.14	UVR	3
PF01205.14	UPF0029	3
PF02254.13	TrkA_N	3
PF13374.1	TPR_10	3
PF07202.8	Tcp10_C	3
PF00464.14	SHMT	3
PF04091.7	Sec15	3

PF14259.1	RRM_6	3
PF08646.5	Rep_fac-A_C	3
PF10262.4	Rdx	3
PF10409.4	PTEN_C2	3
PF07714.12	Pkinase_Tyr	3
PF00433.19	Pkinase_C	3
PF02887.11	PK_C	3
PF00224.16	PK	3
PF01433.15	Peptidase_M1	3
PF05048.8	NosD	3
PF00175.16	NAD_binding_1	3
PF06017.8	Myosin_TH1	3
PF09139.6	Mmp37	3
PF01875.12	Memo	3
PF02373.17	JmjC	3
PF08412.5	Ion_trans_N	3
PF00012.15	HSP70	3
PF00505.14	HMG_box	3
PF00328.17	His_Phos_2	3
PF00632.20	HECT	3
PF00009.22	GTP_EFTU	3
PF00982.16	Glyco_transf_20	3
PF02212.13	GED	3
PF01363.16	FYVE	3
PF01846.14	FF	3
PF01794.14	Ferric_reduct	3
PF12807.2	eIF3_p135	3
PF02319.15	E2F_TDP	3
PF01031.15	Dynamin_M	3
PF02714.10	DUF221	3
PF03455.14	dDENN	3
PF01266.19	DAO	3
PF10557.4	Cullin_Nedd8	3
PF06957.6	COPI_C	3
PF13236.1	CLU	3
PF10607.4	CLTH	3
PF01644.12	Chitin_synth_1	3
PF00686.14	CBM_20	3
PF00339.24	Arrestin_N	3
PF00501.23	AMP-binding	3
PF14739.1	DUF4472	2
PF00569.12	ZZ	2
PF13445.1	zf-RING_UBOX	2
PF00641.13	zf-RanBP	2
PF02891.15	zf-MIZ	2
PF14260.1	zf-C4pol	2

PF03366.11	YEATS	2
PF00752.12	XPG_N	2
PF08774.6	VRR_NUC	2
PF00790.14	VHS	2
PF10595.4	UPF0564	2
PF00789.15	UBX	2
PF00240.18	ubiquitin	2
PF02134.16	UBACT	2
PF00133.17	tRNA-synt_1	2
PF01336.20	tRNA_anti-codon	2
PF13868.1	Trichoplein	2
PF02358.11	Trehalose_PPase	2
PF13848.1	Thioredoxin_6	2
PF01925.14	TauE	2
PF02383.13	Syja_N	2
PF10266.4	Strumpellin	2
PF01852.14	START	2
PF07228.7	SpoIIE	2
PF09335.6	SNARE_assoc	2
PF01713.16	Smr	2
PF01380.17	SIS	2
PF02146.12	SIR2	2
PF00856.23	SET	2
PF00735.13	Septin	2
PF01365.16	RYDR_ITPR	2
PF08454.6	RIH_assoc	2
PF14666.1	RICTOR_M	2
PF09409.5	PUB	2
PF01416.15	PseudoU_synth_1	2
PF01619.13	Pro_dh	2
PF14844.1	PH_BEACH	2
PF14593.1	PH_3	2
PF00162.14	PGK	2
PF00326.16	Peptidase_S9	2
PF07998.6	Peptidase_M54	2
PF01434.13	Peptidase_M41	2
PF00644.15	PARP	2
PF04926.10	PAP_RNA-bind	2
PF03828.14	PAP_assoc	2
PF07034.6	ORC3_N	2
PF14775.1	NYD-SP28_assoc	2
PF01189.12	Nol1_Nop2_Fmu	2
PF00999.16	Na_H_Exchanger	2
PF01699.19	Na_Ca_ex	2
PF06602.9	Myotub-related	2
PF05192.13	MutS_III	2

PF04117.7	Mpv17_PMP22	2
PF08569.6	Mo25	2
PF02854.14	MIF4G	2
PF08241.7	Methyltransf_11	2
PF02820.13	MBT	2
PF00917.21	MATH	2
PF01554.13	MatE	2
PF00390.14	malic	2
PF13504.1	LRR_7	2
PF13385.1	Laminin_G_3	2
PF12706.2	Lactamase_B_2	2
PF00467.24	KOW	2
PF13964.1	Kelch_6	2
PF12309.3	KBP_C	2
PF02375.12	JmjN	2
PF01398.16	JAB	2
PF03810.14	IBN_N	2
PF00447.12	HSF_DNA-bind	2
PF00046.24	Homeobox	2
PF02985.17	HEAT	2
PF03144.20	GTP_EFTU_D2	2
PF00043.20	GST_C	2
PF02893.15	GRAM	2
PF11904.3	GPCR_chapero_1	2
PF00462.19	Glutaredoxin	2
PF02685.11	Glucokinase	2
PF01590.21	GAF	2
PF02687.16	FtsX	2
PF00258.20	Flavodoxin_1	2
PF02181.18	FH2	2
PF02259.18	FAT	2
PF00667.15	FAD_binding_1	2
PF11838.3	ERAP1_C	2
PF01105.19	EMP24_GP25L	2
PF00350.18	Dynamin_N	2
PF05277.7	DUF726	2
PF05018.8	DUF667	2
PF15239.1	DUF4586	2
PF12394.3	DUF3657	2
PF11618.3	DUF3250	2
PF09320.6	DUF1977	2
PF08590.5	DUF1771	2
PF14671.1	DSPn	2
PF05186.8	Dpy-30	2
PF08781.5	DP	2
PF04118.9	Dopey_N	2

PF00684.14	DnaJ_CXXCXGXG	2
PF03104.14	DNA_pol_B_exo1	2
PF00136.16	DNA_pol_B	2
PF01612.15	DNA_pol_A_exo1	2
PF08385.7	DHC_N1	2
PF06733.10	DEAD_2	2
PF00781.19	DAGK_cat	2
PF01467.21	CTP_transf_2	2
PF00118.19	Cpn60_TCP1	2
PF00188.21	CAP	2
PF04857.15	CAF1	2
PF00170.16	bZIP_1	2
PF10996.3	Beta-Casp	2
PF13229.1	Beta_helix	2
PF14781.1	BBS2_N	2
PF14783.1	BBS2_Mid	2
PF14782.1	BBS2_C	2
PF01145.20	Band_7	2
PF04377.10	ATE_C	2
PF00026.18	Asp	2
PF02752.17	Arrestin_C	2
PF11527.3	ARL2_Bind_BART	2
PF04108.7	APG17	2
PF13857.1	Ank_5	2
PF13606.1	Ank_3	2
PF01425.16	Amidase	2
PF14469.1	AKAP28	2
PF00406.17	ADK	2
PF00106.20	adh_short	2
PF02770.14	Acyl-CoA_dh_M	2
PF13508.1	Acetyltransf_7	2
PF13000.2	Acatn	2
PF12697.2	Abhydrolase_6	2
PF13238.1	AAA_18	2
PF02872.13	5_nucleotid_C	2
PF00173.23	Cyt-b5	1
PF00534.15	Glycos_transf_1	1
PF00561.15	Abhydrolase_1	1
PF00929.19	RNase_T	1
PF00994.19	MoCF_biosynth	1
PF01648.15	ACPS	1
PF01687.12	Flavokinase	1
PF01753.13	zf-MYND	1
PF01757.17	Acyl_transf_3	1
PF01764.20	Lipase_3	1
PF02170.17	PAZ	1

PF02171.12	Piwi	1
PF02197.12	RIIa	1
PF02207.15	zf-UBR	1
PF02798.15	GST_N	1
PF02902.14	Peptidase_C48	1
PF02928.11	zf-C5HC2	1
PF02984.14	Cyclin_C	1
PF03351.12	DOMON	1
PF03453.12	MoeA_N	1
PF03454.10	MoeA_C	1
PF03798.11	TRAM_LAG1_CLN8	1
PF04083.11	Abhydro_lipase	1
PF04101.11	Glyco_tran_28_C	1
PF05030.7	SSXT	1
PF06432.6	GPI2	1
PF08059.8	SEP	1
PF08323.6	Glyco_transf_5	1
PF08542.6	Rep_fac_C	1
PF11934.3	DUF3452	1
PF13177.1	DNA_pol3_delta2	1
PF14749.1	Acyl-CoA_ox_N	1
PF14835.1	zf-RING_6	1
PF14634.1	zf-RING_5	1
PF12874.2	zf-met	1
PF13832.1	zf-HC5HC2H_2	1
PF13917.1	zf-CCHC_3	1
PF13696.1	zf-CCHC_2	1
PF01396.14	zf-C4_Topoisom	1
PF13923.1	zf-C3HC4_2	1
PF08265.6	YL1_C	1
PF00867.13	XPG_I	1
PF03007.11	WES_acyltransf	1
PF14745.1	WASH-7_N	1
PF14744.1	WASH-7_mid	1
PF14746.1	WASH-7_C	1
PF13519.1	VWA_2	1
PF05008.10	V-SNARE	1
PF02204.13	VPS9	1
PF07928.7	Vps54	1
PF04100.7	Vps53_N	1
PF04129.7	Vps52	1
PF08700.6	Vps51	1
PF09336.5	Vps4_C	1
PF09454.5	Vps23_core	1
PF04841.8	Vps16_N	1
PF04840.7	Vps16_C	1

PF08487.5	VIT	1
PF11916.3	Vac14_Fig4_bd	1
PF08625.6	Utp13	1
PF00179.21	UQ_con	1
PF10408.4	Ufd2P_core	1
PF03152.9	UFD1	1
PF05743.8	UEV	1
PF01704.13	UDPGP	1
PF03456.13	uDENN	1
PF09358.5	UBA_e1_C	1
PF14555.1	UBA_4	1
PF11717.3	Tudor-knot	1
PF06011.7	TRP	1
PF00152.15	tRNA-synt_2	1
PF00749.16	tRNA-synt_1c	1
PF00579.20	tRNA-synt_1b	1
PF04073.10	tRNA_edit	1
PF12739.2	TRAPPC-Trs85	1
PF13176.1	TPR_7	1
PF02919.10	Topoisom_I_N	1
PF01028.15	Topoisom_I	1
PF14370.1	Topo_C_assoc	1
PF07810.8	TMC	1
PF13905.1	Thioredoxin_8	1
PF00899.16	ThiF	1
PF02882.14	THF_DHG_CYH_C	1
PF00763.18	THF_DHG_CYH	1
PF02291.10	TFIID-31kDa	1
PF12612.3	TFCD_C	1
PF03849.9	Tfb2	1
PF10193.4	Telomere_reg-2	1
PF12009.3	Telomerase_RBD	1
PF03645.8	Tctex-1	1
PF07986.7	TBCC	1
PF08231.7	SYF2	1
PF03015.14	Sterile	1
PF00319.13	SRF-TF	1
PF00494.14	SQS_PSY	1
PF03439.8	Spt5-NGN	1
PF00588.14	SpoU_methylase	1
PF04855.7	SNF5	1
PF00209.13	SNF	1
PF05739.14	SNARE	1
PF14938.1	SNAP	1
PF02463.14	SMC_N	1
PF09111.5	SLIDE	1

PF06046.8	Sec6	1
PF04811.10	Sec23_trunk	1
PF08033.7	Sec23_BS	1
PF00995.18	Sec1	1
PF05285.7	SDA1	1
PF03803.10	Scramblase	1
PF04499.10	SAPS	1
PF07647.12	SAM_2	1
PF00536.25	SAM_1	1
PF03399.11	SAC3_GANP	1
PF02265.11	S1-P1_nuclease	1
PF05773.17	RWD	1
PF00078.22	RVT_1	1
PF13967.1	RSN1_TM	1
PF13893.1	RRM_5	1
PF14700.1	RPOL_N	1
PF01876.11	RNase_P_p30	1
PF01351.13	RNase_HII	1
PF00940.14	RNA_pol	1
PF09414.5	RNA_ligase	1
PF07521.7	RMMBL	1
PF14668.1	RICTOR_V	1
PF01778.12	Ribosomal_L28e	1
PF00636.21	Ribonuclease_3	1
PF00581.15	Rhodanese	1
PF08519.7	RFC1	1
PF04851.10	ResIII	1
PF05183.7	RdRP	1
PF12234.3	Rav1p_C	1
PF14663.1	RasGEF_N_2	1
PF08771.6	Rapamycin_bind	1
PF06098.6	Radial_spoke_3	1
PF08423.6	Rad51	1
PF03215.10	Rad17	1
PF07992.9	Pyr_redox_2	1
PF14306.1	PUA_2	1
PF02666.10	PS_Dcarbxylase	1
PF08799.6	PRP4	1
PF03371.10	PRP38	1
PF09785.4	Prp31_C	1
PF06991.6	Prp19_bind	1
PF02840.10	Prp18	1
PF00432.16	Prenyltrans	1
PF14895.1	PPPI_inhib	1
PF02765.12	POT1	1
PF03900.10	Porphobil_deamC	1

PF01379.15	Porphobil_deam	1
PF00348.12	polyprenyl_synt	1
PF13091.1	PLDc_2	1
PF08190.7	PIH1	1
PF05721.8	PhyH	1
PF13831.1	PHD_2	1
PF00628.24	PHD	1
PF04757.9	Pex2_Pex12	1
PF08634.5	Pet127	1
PF01435.13	Peptidase_M48	1
PF00557.19	Peptidase_M24	1
PF00675.15	Peptidase_M16	1
PF13529.1	Peptidase_C39_2	1
PF01088.16	Peptidase_C12	1
PF13599.1	Pentapeptide_4	1
PF00805.17	Pentapeptide	1
PF01804.13	Penicil_amidase	1
PF00564.19	PB1	1
PF05028.9	PARG_cat	1
PF14874.1	PapD-like	1
PF14738.1	PaaSYMP	1
PF00067.17	p450	1
PF04045.9	P34-Arc	1
PF05517.7	p25-alpha	1
PF02338.14	OTU	1
PF14418.1	OHA	1
PF03800.9	Nuf2	1
PF00293.23	NUDIX	1
PF08159.7	NUC153	1
PF08158.7	NUC130_3NT	1
PF10358.4	NT-C2	1
PF08060.8	NOSIC	1
PF01798.13	Nop	1
PF08155.6	NOGCT	1
PF06858.9	NOG1	1
PF07540.6	NOC3p	1
PF04981.8	NMD3	1
PF09797.4	NatB_MDM20	1
PF07993.7	NAD_binding_4	1
PF15508.1	NAAA-beta	1
PF00063.16	Myosin_head	1
PF14646.1	MYCBPAP	1
PF11831.3	Myb_Cef	1
PF11978.3	MVP_shoulder	1
PF01624.15	MutS_I	1
PF03972.9	MmgE_PrpD	1

PF13012.1	MitMem_reg	1
PF00230.15	MIP	1
PF09088.6	MIF4G_like	1
PF02636.12	Methyltransf_28	1
PF13489.1	Methyltransf_23	1
PF12847.2	Methyltransf_18	1
PF08711.6	Med26	1
PF00493.18	MCM	1
PF05292.6	MCD	1
PF04188.8	Mannosyl_trans2	1
PF03949.10	Malic_M	1
PF04874.9	Mak16	1
PF15294.1	Leu_zip	1
PF07766.8	LETM1	1
PF06421.7	LepA_C	1
PF15619.1	Lebercilin	1
PF05147.8	LANC_like	1
PF03731.10	Ku_N	1
PF03730.9	Ku_C	1
PF02735.11	Ku	1
PF09762.4	KOG2701	1
PF12423.3	KIF1B	1
PF14714.1	KH_dom-like	1
PF13925.1	Katanin_con80	1
PF05804.7	KAP	1
PF01156.14	IU_nuc_hydro	1
PF06246.7	Isy1	1
PF08709.6	Ins145_P3_rec	1
PF05770.6	Ins134_P3_kin	1
PF01652.13	IF4E	1
PF08461.5	HTH_12	1
PF01627.18	Hpt	1
PF00368.13	HMG-CoA_red	1
PF09011.5	HMG_box_2	1
PF03006.15	HlyIII	1
PF14520.1	HHH_5	1
PF13625.1	Helicase_C_3	1
PF13307.1	Helicase_C_2	1
PF13646.1	HEAT_2	1
PF01966.17	HD	1
PF14661.1	HAUS6_N	1
PF01725.11	Ham1p_like	1
PF08152.7	GUCT	1
PF03199.10	GSH_synthase	1
PF03917.12	GSH_synth_ATP	1
PF07919.7	Gryzun	1

PF01465.15	GRIP	1
PF10192.4	GpcrRhopsn4	1
PF12656.2	G-patch_2	1
PF00274.14	Glycolytic	1
PF01074.17	Glyco_hydro_38	1
PF04960.10	Glutaminase	1
PF04828.9	GFA	1
PF00626.17	Gelsolin	1
PF03009.12	GDPD	1
PF01571.16	GCV_T	1
PF03074.11	GCS	1
PF13537.1	GATase_7	1
PF03127.9	GAT	1
PF02187.12	GAS2	1
PF04410.9	Gar1	1
PF13492.1	GAF_3	1
PF03630.9	Fumble	1
PF11817.3	Foie-gras_1	1
PF00041.16	fn3	1
PF00254.23	FKBP_C	1
PF00630.14	Filamin	1
PF08151.7	FerI	1
PF00646.28	F-box	1
PF00487.19	FA_desaturase	1
PF00754.20	F5_F8_type_C	1
PF10256.4	Erf4	1
PF02732.10	ERCC4	1
PF00113.17	Enolase_C	1
PF03764.13	EFG_IV	1
PF14492.1	EFG_II	1
PF00679.19	EFG_C	1
PF00676.15	E1_dh	1
PF13815.1	Dzip-like_N	1
PF04912.9	Dynamitin	1
PF00692.14	dUTPase	1
PF06067.6	DUF932	1
PF05664.6	DUF810	1
PF04950.7	DUF663	1
PF15261.1	DUF4591	1
PF14908.1	DUF4496	1
PF14858.1	DUF4486	1
PF14846.1	DUF4485	1
PF14644.1	DUF4456	1
PF14643.1	DUF4455	1
PF14237.1	DUF4339	1
PF13821.1	DUF4187	1

PF12842.2	DUF3819	1
PF10300.4	DUF3808	1
PF12166.3	DUF3595	1
PF12066.3	DUF3546	1
PF12022.3	DUF3510	1
PF11931.3	DUF3449	1
PF10474.4	DUF2451	1
PF10475.4	DUF2450	1
PF02995.12	DUF229	1
PF08217.6	DUF1712	1
PF08014.6	DUF1704	1
PF07713.8	DUF1604	1
PF06650.7	DUF1162	1
PF06565.7	DUF1126	1
PF06245.6	DUF1015	1
PF08123.8	DOT1	1
PF00476.15	DNA_pol_A	1
PF01119.14	DNA_mis_repair	1
PF04675.9	DNA_ligase_A_N	1
PF01068.16	DNA_ligase_A_M	1
PF04679.10	DNA_ligase_A_C	1
PF06584.8	DIRP	1
PF00186.14	DHFR_1	1
PF10407.4	Cytokin_check_N	1
PF04676.9	CwfJ_C_2	1
PF04677.10	CwfJ_C_1	1
PF01083.17	Cutinase	1
PF04145.10	Ctr	1
PF04442.9	CtaG_Cox11	1
PF00313.17	CSD	1
PF03765.10	CRAL_TRIO_N	1
PF03178.10	CPSF_A	1
PF08695.5	Coa1	1
PF02515.12	CoA_transf_3	1
PF12717.2	Cnd1	1
PF02487.12	CLN3	1
PF01086.12	Clathrin_lg_ch	1
PF01214.13	CK_II_beta	1
PF00385.19	Chromo	1
PF12624.2	Chorein_N	1
PF04968.7	CHORD	1
PF09295.5	ChAPs	1
PF00307.26	CH	1
PF15627.1	CEP76-C2	1
PF05995.7	CDO_I	1
PF05179.9	CDC73	1

PF06203.9	CCT	1
PF15625.1	CC2D2AN-C2	1
PF00571.23	CBS	1
PF03914.12	CBF	1
PF00690.21	Cation_ATPase_N	1
PF08683.6	CAMSAP_CKK	1
PF12265.3	CAF1C_H4-bd	1
PF09736.4	Bud13	1
PF04427.13	Brix	1
PF03099.14	BPL_LplA_LipB	1
PF00364.17	Biotin_lipoyl	1
PF09794.4	Avl9	1
PF06644.6	ATP11	1
PF01171.15	ATP_bind_3	1
PF09333.6	ATG_C	1
PF04376.8	ATE_N	1
PF01400.19	Astacin	1
PF00710.15	Asparaginase	1
PF04729.8	ASF1_hist_chap	1
PF02374.10	ArsA_ATPase	1
PF04959.8	ARS2	1
PF10208.4	Armet	1
PF03308.11	ArgK	1
PF10351.4	Apt1	1
PF01583.15	APS_kinase	1
PF01871.12	AMMECR1	1
PF09261.6	Alpha-mann_mid	1
PF00171.17	Aldedh	1
PF11559.3	ADIP	1
PF01602.15	Adaptin_N	1
PF00928.16	Adap_comp_sub	1
PF02771.11	Acyl-CoA_dh_N	1
PF00441.19	Acyl-CoA_dh_1	1
PF01756.14	ACOX	1
PF00583.19	Acetyltransf_1	1
PF12698.2	ABC2_membrane_3	1
PF12430.3	ABA_GPCR	1
PF13521.1	AAA_28	1
PF13207.1	AAA_17	1
PF01073.14	3Beta_HSD	1
PF00198.18	2-oxoacid_dh	1

Table S5. Distribution of PRF genes in KEGG pathway database

Entry	Name	Occurrence
ko01100	Metabolic pathways	68
ko01110	Biosynthesis of secondary metabolites	27
ko01130	Biosynthesis of antibiotics	22
ko04151	PI3K-Akt signaling pathway	18
ko03040	Spliceosome	16
ko04068	FoxO signaling pathway	15
ko04910	Insulin signaling pathway	15
ko00230	Purine metabolism	14
ko04071	Sphingolipid signaling pathway	14
ko04114	Oocyte meiosis	14
ko04144	Endocytosis	14
ko04152	AMPK signaling pathway	14
ko04921	Oxytocin signaling pathway	14
ko04931	Insulin resistance	14
ko03420	Nucleotide excision repair	13
ko04024	cAMP signaling pathway	13
ko04110	Cell cycle	13
ko04113	Meiosis - yeast	13
ko05200	Pathways in cancer	13
ko05205	Proteoglycans in cancer	13
ko01120	Microbial metabolism in diverse environments	12
ko04010	MAPK signaling pathway	12
ko04022	cGMP-PKG signaling pathway	12
ko04111	Cell cycle - yeast	12
ko04141	Protein processing in endoplasmic reticulum	12
ko04150	mTOR signaling pathway	12
ko04261	Adrenergic signaling in cardiomyocytes	12
ko05164	Influenza A	12
ko05166	HTLV-I infection	12
ko05169	Epstein-Barr virus infection	12
ko05203	Viral carcinogenesis	12
ko03015	mRNA surveillance pathway	11
ko04070	Phosphatidylinositol signaling system	11
ko04120	Ubiquitin mediated proteolysis	11
ko04310	Wnt signaling pathway	11
ko04666	Fc gamma R-mediated phagocytosis	11
ko04728	Dopaminergic synapse	11
ko04810	Regulation of actin cytoskeleton	11
ko04919	Thyroid hormone signaling pathway	11
ko05231	Choline metabolism in cancer	11
ko00562	Inositol phosphate metabolism	10
ko03008	Ribosome biogenesis in eukaryotes	10
ko04145	Phagosome	10
ko04270	Vascular smooth muscle contraction	10

ko04720	Long-term potentiation	10
ko04722	Neurotrophin signaling pathway	10
ko04914	Progesterone-mediated oocyte maturation	10
ko05230	Central carbon metabolism in cancer	10
ko01200	Carbon metabolism	9
ko03430	Mismatch repair	9
ko04020	Calcium signaling pathway	9
ko04066	HIF-1 signaling pathway	9
ko04072	Phospholipase D signaling pathway	9
ko04510	Focal adhesion	9
ko04540	Gap junction	9
ko04611	Platelet activation	9
ko04725	Cholinergic synapse	9
ko05152	Tuberculosis	9
ko05162	Measles	9
ko05206	MicroRNAs in cancer	9
ko05215	Prostate cancer	9
ko02020	Two-component system	8
ko03013	RNA transport	8
ko03030	DNA replication	8
ko04012	ErbB signaling pathway	8
ko04062	Chemokine signaling pathway	8
ko04530	Tight junction	8
ko04750	Inflammatory mediator regulation of TRP channels	8
ko04915	Estrogen signaling pathway	8
ko04920	Adipocytokine signaling pathway	8
ko04922	Glucagon signaling pathway	8
ko05160	Hepatitis C	8
ko05168	Herpes simplex infection	8
ko05213	Endometrial cancer	8
ko00240	Pyrimidine metabolism	7
ko01230	Biosynthesis of amino acids	7
ko03018	RNA degradation	7
ko03460	Fanconi anemia pathway	7
ko04015	Rap1 signaling pathway	7
ko04146	Peroxisome	7
ko04380	Osteoclast differentiation	7
ko04660	T cell receptor signaling pathway	7
ko04662	B cell receptor signaling pathway	7
ko04724	Glutamatergic synapse	7
ko04912	GnRH signaling pathway	7
ko04925	Aldosterone synthesis and secretion	7
ko05161	Hepatitis B	7
ko05210	Colorectal cancer	7
ko05214	Glioma	7
ko00010	Glycolysis / Gluconeogenesis	6

ko04014	Ras signaling pathway	6
ko04140	Regulation of autophagy	6
ko04142	Lysosome	6
ko04340	Hedgehog signaling pathway	6
ko04370	VEGF signaling pathway	6
ko04390	Hippo signaling pathway	6
ko04391	Hippo signaling pathway - fly	6
ko04664	Fc epsilon RI signaling pathway	6
ko04668	TNF signaling pathway	6
ko04713	Circadian entrainment	6
ko04726	Serotonergic synapse	6
ko04911	Insulin secretion	6
ko04916	Melanogenesis	6
ko04923	Regulation of lipolysis in adipocytes	6
ko04930	Type II diabetes mellitus	6
ko04932	Non-alcoholic fatty liver disease (NAFLD)	6
ko04962	Vasopressin-regulated water reabsorption	6
ko04970	Salivary secretion	6
ko05010	Alzheimer's disease	6
ko05016	Huntington's disease	6
ko05132	Salmonella infection	6
ko05145	Toxoplasmosis	6
ko05221	Acute myeloid leukemia	6
ko00071	Fatty acid degradation	5
ko00520	Amino sugar and nucleotide sugar metabolism	5
ko00900	Terpenoid backbone biosynthesis	5
ko00970	Aminoacyl-tRNA biosynthesis	5
ko03022	Basal transcription factors	5
ko03440	Homologous recombination	5
ko04210	Apoptosis	5
ko04350	TGF-beta signaling pathway	5
ko04550	Signaling pathways regulating pluripotency of stem cells	5
ko04723	Retrograde endocannabinoid signaling	5
ko04730	Long-term depression	5
ko04917	Prolactin signaling pathway	5
ko04924	Renin secretion	5
ko04972	Pancreatic secretion	5
ko04976	Bile secretion	5
ko05031	Amphetamine addiction	5
ko05032	Morphine addiction	5
ko05034	Alcoholism	5
ko05110	Vibrio cholerae infection	5
ko05130	Pathogenic Escherichia coli infection	5
ko05131	Shigellosis	5
ko05134	Legionellosis	5
ko05212	Pancreatic cancer	5

ko05218	Melanoma	5
ko05223	Non-small cell lung cancer	5
ko00260	Glycine, serine and threonine metabolism	4
ko00310	Lysine degradation	4
ko00480	Glutathione metabolism	4
ko00500	Starch and sucrose metabolism	4
ko01212	Fatty acid metabolism	4
ko03320	PPAR signaling pathway	4
ko04115	p53 signaling pathway	4
ko04520	Adherens junction	4
ko04620	Toll-like receptor signaling pathway	4
ko04650	Natural killer cell mediated cytotoxicity	4
ko04670	Leukocyte transendothelial migration	4
ko04721	Synaptic vesicle cycle	4
ko04727	GABAergic synapse	4
ko04745	Phototransduction - fly	4
ko04960	Aldosterone-regulated sodium reabsorption	4
ko04971	Gastric acid secretion	4
ko05020	Prion diseases	4
ko05100	Bacterial invasion of epithelial cells	4
ko05142	Chagas disease (American trypanosomiasis)	4
ko05211	Renal cell carcinoma	4
ko05220	Chronic myeloid leukemia	4
ko05222	Small cell lung cancer	4
ko00250	Alanine, aspartate and glutamate metabolism	3
ko00270	Cysteine and methionine metabolism	3
ko00280	Valine, leucine and isoleucine degradation	3
ko00330	Arginine and proline metabolism	3
ko00360	Phenylalanine metabolism	3
ko00510	N-Glycan biosynthesis	3
ko00513	Various types of N-glycan biosynthesis	3
ko00620	Pyruvate metabolism	3
ko00670	One carbon pool by folate	3
ko00680	Methane metabolism	3
ko00860	Porphyrin and chlorophyll metabolism	3
ko02010	ABC transporters	3
ko03410	Base excision repair	3
ko04075	Plant hormone signal transduction	3
ko04320	Dorso-ventral axis formation	3
ko04360	Axon guidance	3
ko04626	Plant-pathogen interaction	3
ko04630	Jak-STAT signaling pathway	3
ko04710	Circadian rhythm	3
ko04740	Olfactory transduction	3
ko04918	Thyroid hormone synthesis	3
ko04961	Endocrine and other factor-regulated calcium reabsorption	3

ko05012	Parkinson's disease	3
ko05146	Amoebiasis	3
ko05414	Dilated cardiomyopathy	3
ko00061	Fatty acid biosynthesis	2
ko00130	Ubiquinone and other terpenoid-quinone biosynthesis	2
ko00190	Oxidative phosphorylation	2
ko00220	Arginine biosynthesis	2
ko00350	Tyrosine metabolism	2
ko00380	Tryptophan metabolism	2
ko00400	Phenylalanine, tyrosine and tryptophan biosynthesis	2
ko00401	Novobiocin biosynthesis	2
ko00450	Selenocompound metabolism	2
ko00564	Glycerophospholipid metabolism	2
ko00600	Sphingolipid metabolism	2
ko00710	Carbon fixation in photosynthetic organisms	2
ko00903	Limonene and pinene degradation	2
ko00950	Isoquinoline alkaloid biosynthesis	2
ko00960	Tropane, piperidine and pyridine alkaloid biosynthesis	2
ko03050	Proteasome	2
ko03450	Non-homologous end-joining	2
ko04011	MAPK signaling pathway - yeast	2
ko04013	MAPK signaling pathway - fly	2
ko04064	NF-kappa B signaling pathway	2
ko04330	Notch signaling pathway	2
ko04621	NOD-like receptor signaling pathway	2
ko04711	Circadian rhythm - fly	2
ko04712	Circadian rhythm - plant	2
ko04913	Ovarian steroidogenesis	2
ko04973	Carbohydrate digestion and absorption	2
ko05014	Amyotrophic lateral sclerosis (ALS)	2
ko05120	Epithelial cell signaling in Helicobacter pylori infection	2
ko05133	Pertussis	2
ko05204	Chemical carcinogenesis	2
ko05216	Thyroid cancer	2
ko05219	Bladder cancer	2
ko05410	Hypertrophic cardiomyopathy (HCM)	2
ko00020	Citrate cycle (TCA cycle)	1
ko00030	Pentose phosphate pathway	1
ko00040	Pentose and glucuronate interconversions	1
ko00051	Fructose and mannose metabolism	1
ko00052	Galactose metabolism	1
ko00053	Ascorbate and aldarate metabolism	1
ko00062	Fatty acid elongation	1
ko00073	Cutin, suberine and wax biosynthesis	1
ko00100	Steroid biosynthesis	1
ko00261	Monobactam biosynthesis	1

ko00311	Penicillin and cephalosporin biosynthesis	1
ko00340	Histidine metabolism	1
ko00363	Bisphenol degradation	1
ko00410	beta-Alanine metabolism	1
ko00430	Taurine and hypotaurine metabolism	1
ko00440	Phosphonate and phosphinate metabolism	1
ko00460	Cyanoamino acid metabolism	1
ko00471	D-Glutamine and D-glutamate metabolism	1
ko00511	Other glycan degradation	1
ko00514	Other types of O-glycan biosynthesis	1
ko00521	Streptomycin biosynthesis	1
ko00524	Butirosin and neomycin biosynthesis	1
ko00550	Peptidoglycan biosynthesis	1
ko00561	Glycerolipid metabolism	1
ko00563	Glycosylphosphatidylinositol(GPI)-anchor biosynthesis	1
ko00592	alpha-Linolenic acid metabolism	1
ko00604	Glycosphingolipid biosynthesis - ganglio series	1
ko00624	Polycyclic aromatic hydrocarbon degradation	1
ko00625	Chloroalkane and chloroalkene degradation	1
ko00627	Aminobenzoate degradation	1
ko00630	Glyoxylate and dicarboxylate metabolism	1
ko00720	Carbon fixation pathways in prokaryotes	1
ko00740	Riboflavin metabolism	1
ko00760	Nicotinate and nicotinamide metabolism	1
ko00770	Pantothenate and CoA biosynthesis	1
ko00785	Lipoic acid metabolism	1
ko00790	Folate biosynthesis	1
ko00830	Retinol metabolism	1
ko00910	Nitrogen metabolism	1
ko00920	Sulfur metabolism	1
ko00945	Stilbenoid, diarylheptanoid and gingerol biosynthesis	1
ko00980	Metabolism of xenobiotics by cytochrome P450	1
ko00982	Drug metabolism - cytochrome P450	1
ko00983	Drug metabolism - other enzymes	1
ko01040	Biosynthesis of unsaturated fatty acids	1
ko01210	2-Oxocarboxylic acid metabolism	1
ko04122	Sulfur relay system	1
ko04130	SNARE interactions in vesicular transport	1
ko04612	Antigen processing and presentation	1
ko04622	RIG-I-like receptor signaling pathway	1
ko04742	Taste transduction	1
ko04940	Type I diabetes mellitus	1
ko04964	Proximal tubule bicarbonate reclamation	1
ko04966	Collecting duct acid secretion	1
ko04974	Protein digestion and absorption	1
ko04977	Vitamin digestion and absorption	1

ko04978	Mineral absorption	1
ko05030	Cocaine addiction	1
ko05140	Leishmaniasis	1
ko05202	Transcriptional misregulation in cancer	1
ko05217	Basal cell carcinoma	1
ko05323	Rheumatoid arthritis	1
ko05412	Arrhythmogenic right ventricular cardiomyopathy (ARVC)	1
ko05416	Viral myocarditis	1

Table S6. The list of putative tRNAs of *Euplotes octocarinatus*

Sequence Name	tRNA	tRNA Begin	Bounds End	tRNA Type	Anti Codon	Intron Begin	Bounds End	Cove Score
Contig2146	1	2168	2239	Thr	TGT	0	0	45
Contig2236	1	2659	2588	Thr	AGT	0	0	77.45
Contig2371	1	343	415	Val	AAC	0	0	76.18
Contig2971	1	1313	1243	Phe	GAA	0	0	42.05
Contig3018	1	782	906	Lys	TTT	819	871	37.41
Contig3410	1	787	858	Phe	GAA	0	0	54.7
Contig6440	1	798	726	Arg	TCG	0	0	68.88
Contig7341	1	145	225	Leu	CAA	0	0	69.41
Contig7673	1	487	559	Lys	CTT	0	0	85.42
Contig7885	1	671	743	Arg	CCG	0	0	60.52
Contig8078	1	1412	1483	Gly	TCC	0	0	83.19
Contig8190	1	307	378	Lys	TTT	0	0	87.11
Contig8502	1	831	902	Gly	TCC	0	0	83.19
Contig8850	1	477	548	Cys	GCA	0	0	76.11
Contig10439	1	557	628	Ala	TGC	0	0	74.34
Contig10641	1	172	94	Leu	AAG	0	0	70.46
Contig12814	1	457	528	Gln	TTG	0	0	71.49
Contig13077	1	283	353	Undet	???	0	0	59.95
Contig16875	1	168	97	Gln	CTG	0	0	68.88
Contig19075	1	1908	1979	Phe	GAA	0	0	56.73
Contig20134	1	731	809	Leu	TAG	0	0	74.41
Contig20433	1	320	391	Lys	TTT	0	0	87.11
Contig20964	1	164	93	Pro	CGG	0	0	76.25
Contig20990	1	166	94	Asn	GTT	0	0	79.53
Contig21001	1	229	300	Glu	CTC	0	0	64.99
Contig21002	1	175	104	Met	CAT	0	0	66.22
Contig21004	1	174	102	Val	TAC	0	0	78.31
Contig21006	1	173	102	Asp	GTC	0	0	73.24
Contig21038	1	468	539	SeC	TCA	0	0	63.83
Contig21063	1	481	552	Pro	AGG	0	0	73.86
Contig21088	1	174	102	Glu	CTC	0	0	74.73
Contig21112	1	304	375	Ala	TGC	0	0	74.34
Contig21263	1	191	119	Arg	CCT	0	0	64.53
Contig21285	1	175	94	Leu	AAG	0	0	62.27
Contig21316	1	190	108	Ser	GCT	0	0	74.17
Contig21362	1	200	271	His	GTG	0	0	53.11
Contig24862	1	2215	2137	Leu	CAG	0	0	71.98
Contig25097	1	764	679	Tyr	GTA	727	715	71.93
Contig26740	1	1198	1126	Val	TAC	0	0	52
Contig27720	1	441	523	Leu	TAA	0	0	66.72

Contig29485	1	729	658	Gly	CCC	0	0	78.2
Contig29485	2	183	110	Undet	???	0	0	23.35
Contig29505	1	2229	2307	Leu	AAG	0	0	70.46
Contig29511	1	164	92	Arg	TCG	0	0	35.45
Contig30280	1	20	92	Met	CAT	0	0	75.15
Contig30280	2	999	926	Arg	CCT	0	0	69.01
Contig32200	1	178	93	Tyr	GTA	141	129	71.93
Contig32248	1	324	397	Arg	TCT	0	0	72.04
Contig32382	1	503	432	Lys	TTT	0	0	87.11
Contig32580	1	2205	2127	Leu	CAG	0	0	65.77
Contig33447	1	535	606	Thr	AGT	0	0	77.45
Contig33508	1	265	338	Lys	TTT	0	0	77.3
Contig33553	1	436	365	Glu	TTC	0	0	65.22
Contig33585	1	482	555	Met	CAT	0	0	58.47
Contig33628	1	327	257	Gly	GCC	0	0	62.73
Contig33653	1	173	101	Glu	TTC	0	0	73.11
Contig33667	1	352	283	Undet	???	0	0	42.35
Contig33731	1	158	87	Val	CAC	0	0	50.69
Contig33819	1	155	84	Thr	CGT	0	0	72.5
Contig34213	1	457	528	Val	CAC	0	0	52.69
Contig34254	1	373	453	Leu	TAG	0	0	56.54
Contig34482	1	326	399	Arg	TCT	0	0	72.04
Contig34502	1	382	462	Leu	TAA	0	0	63.26
Contig34518	1	335	407	Trp	CCA	0	0	68.76
Contig34522	1	158	87	Leu	CAA	0	0	54.2
Contig34579	1	149	79	Leu	CAG	0	0	52.31
Contig34628	1	187	115	Arg	ACG	0	0	68.09
Contig34652	1	304	375	Gln	TTG	0	0	71.49
Contig34700	1	319	391	Ala	AGC	0	0	54.02
Contig34704	1	319	391	Ala	AGC	0	0	54.02
Contig34760	1	166	94	Phe	GAA	0	0	70.81
Contig34775	1	179	107	Ile	TAT	0	0	58.85
Contig34792	1	312	384	Trp	CCA	0	0	64.36
Contig34794	1	317	236	Ser	CGA	0	0	84.74
Contig34811	1	293	364	Ala	CGC	0	0	79.61
Contig34821	1	289	370	Ser	TGA	0	0	84.95
Contig34822	1	291	372	Ser	AGA	0	0	82.67
Contig34823	1	166	94	Met	CAT	0	0	73.55
Contig34855	1	162	91	Pro	TGG	0	0	75.72
Contig34863	1	180	109	Thr	TGT	0	0	79.74
Contig34916	1	138	57	Leu	CAG	0	0	64.43
Contig34960	1	283	355	Ile	AAT	0	0	81.9
Contig34977	1	180	109	Thr	TGT	0	0	79.74
Contig34983	1	296	367	Phe	AAA	0	0	76.6

Contig35058	1	273	345	Ile	AAT	0	0	81.9
Contig35093	1	161	89	Ile	TAT	0	0	57.71
Contig35145	1	275	346	Ile	GAT	0	0	64.45
Contig35710	1	108	36	Val	CAC	0	0	80.77
Contig36094	1	143	225	Sup	TTA	180	190	42.04
Contig36987	1	83	154	Lys	TTT	0	0	84.34
Contig37095	1	97	170	Arg	CCG	0	0	59.29
Contig38952	1	89	16	Arg	ACG	0	0	74.62
Contig39938	1	193	113	Sup	TTA	0	0	40.25
Contig40166	1	119	190	Glu	TTC	0	0	67.82
Contig4144533	1	171	85	Tyr	GTA	133	121	68.15

Table S7. Properties of *ab initio* predicted genes

Feature	Number	Mean (bp)	Min (bp)	Max (bp)	%GC
Genes	24,883	1,724	309	18,625	29.1
Exons	82,697	380	3	11,848	31.3
Introns	56,263	189	25	1,127	23.3
CDS	24,883	1,175	213	18,342	31.9
5’upstream of start codon	21,692	372	21	9,043	21.6
3’downstream of stop codon	21,692	331	3	10,489	21.6

Prediction features were obtained for complete non-PRF nanochromosomes (23,220 in total) only. Up- and downstream regions were only determined for single-gene nanochromosomes (21,692 in total). %GC estimates exclude telomeric bases.
